# Trends and Directions of Preference Elicitation and Assessment in Food Science: Single‐, Pair‐, and Multi‐Criteria Ranking Methods

**DOI:** 10.1002/fsn3.70684

**Published:** 2025-07-31

**Authors:** László Sipos, Zsófia Galambosi, Péter Biró, László Csató, Sándor Bozóki

**Affiliations:** ^1^ Institute of Food Science and Technology, Department of Postharvest, Commercial and Sensory Science Hungarian University of Agriculture and Life Sciences Budapest Hungary; ^2^ Centre of Economic and Regional Studies, Hungarian Research Network (HUN‐REN) Institute of Economics Budapest Hungary; ^3^ Institute of Operations Research and Decision Systems, Department of Operations Research and Actuarial Sciences Corvinus University of Budapest Budapest Hungary; ^4^ Institute for Computer Science and Control (SZTAKI), Hungarian Research Network (HUN‐REN), laboratory on Engineering and Management Intelligence, Research Group of Operations Research and Decision Systems Budapest Hungary

**Keywords:** analytic hierarchy process (AHP), balanced incomplete block design (BIBD), multi‐criteria decision making (MCDM), pairwise ranking, sensory food competitions

## Abstract

Different ranking methods have long been used in sensory testing. Their diffusion was mainly due to their simplicity, their standardized methods, their software support, and their wide range of applications. However, a number of research and industrial problems have emerged recently that cannot be adequately solved by the standard approaches, making it necessary to apply and adapt methods from other disciplines. This article discusses the ranking methods of international sensory standardization (single‐, pair‐ranking methods, balanced incomplete block design (BIBD) according to the structure of standardization). We overview ranking methods in international sensory competitions. We evaluate some potential applications of ranking methodological developments pair ranking, Analytic Hierarchy Process (AHP), multi‐criteria decision making (MCDM), graph theory, developed and applied by other disciplines, which are less used in sensory sciences. The principles and applications of these methodologies are summarized, and factors affecting their effectiveness and limitations are discussed. Preference modeling and quantification are key questions of decision and sensory sciences. Both areas require true, unbiased individual responses as well as their appropriate aggregation. Competitions are typically multi‐criteria problems, evaluated by several decision makers. To better understand consumer preferences and to explore complex consumer choices, different methods and software from other disciplines—decision and social choice theory, operations research, economics (e.g., ranking based on pairwise comparisons, AHP, MCDM, graph theory)—are adapted and disseminated. In addition to surveying latest trends, we propose some areas of future research development.

## Graphical abstract

The advantages and limitations of standard ranking techniques are evaluated. Related methodologies from other disciplines have the potential to sensory studies. International food competition rules are heterogeneous and insufficiently specified. Guidance for future research directions in decision methodologies is provided.

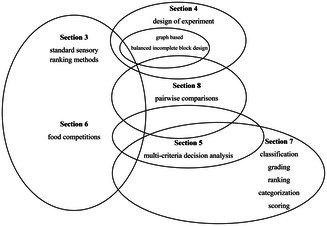

## 1 Introduction

Food testing is usually performed according to standard specifications, under standard conditions, using standard methods. Standards are technical documents, drawn up/approved by a recognized body, which are generally accepted by consensus, cover an activity or its results, and contain general and reproducible rules, guidelines, or characteristics. Standardization in the world is highly diversified, both in time and space (international, regional, national) and in terms of subject matter. Currently, international standards are developed by 832 Technical Committees (TCs) of the 170 member national standards bodies of the International Organization for Standardization (ISO).

Various standard test methods have been developed for the order of preference of foods. The standardization field of the International Organization for Standardization (ISO), Food Products (TC 34), Subcommittee on Sensory (SC 12), covers the vocabulary, general guidelines, selection and training of assessors performing sensory testing, and the methodology for performing the various tests. The secretariat of the subcommittee is currently provided by Association française de normalization (AFNOR). ISO has developed more than 25,000 international standards, of which the Sensory Subcommittee (ISO/TC 34/SC 12) has 39 published ISO standards and three standards under development. The international nature of the Sensory Subcommittee (ISO/TC 34/SC 12) is evidenced by the 29 participants (national standards bodies) status, 24 observer status on all continents, and seven working groups. The international harmonized section codes show exactly which subsection of the standardization is in which section (detailed information is available on the official ISO website: https://www.iso.org/home.html).

Besides the dominance of standard methods, there are further statistical methods for evaluating ranking tests. Ranking evaluations are based exclusively on a predefined sensory attribute or preference value, but it may be necessary to provide a multi‐criteria ranking of products, combined with other statistical methods. In this paper, we aim to provide alternative statistical methods to standard ranking methods, and to combine and identify the possibilities for the development of multi‐criteria ranking methods. We also present and evaluate the ranking methods of standard sensory tests (conditions, assessors, procedures, statistical analysis, results). A specific objective is to analyze the ranking areas of some international sensory competitions. Because of its importance, we aim to present and evaluate methodological innovations developed and applied by other disciplines (decision theory, economics, operations research, social choice), and their methods such as pairwise ranking, Analytic Hierarchy Process (AHP), multi‐criteria decision making (MCDM), graph theory. Some of these novel techniques will probably be endorsed and become standard methodologies in sensory tests in the future; these new methodologies need to be validated and compared with the current standardized methods.

The main contributions of our work to the literature can be summarized as follows: a collection, review, and discussion of standard ranking methods and experimental designs used in practical sensory research, graph visualization of balanced incomplete block design (BIBD) and elicitation of calculations (graph theoretical parameters), a review of sensory‐focused scoring competitions (identification of anomalies), collection of international beverage‐related sensory competitions, and comparison of international wine and olive oil scoring competitions. In addition, a section is dedicated to the identification of future challenges and trends of ranking and assessment in food science.

**FIGURE 1:**
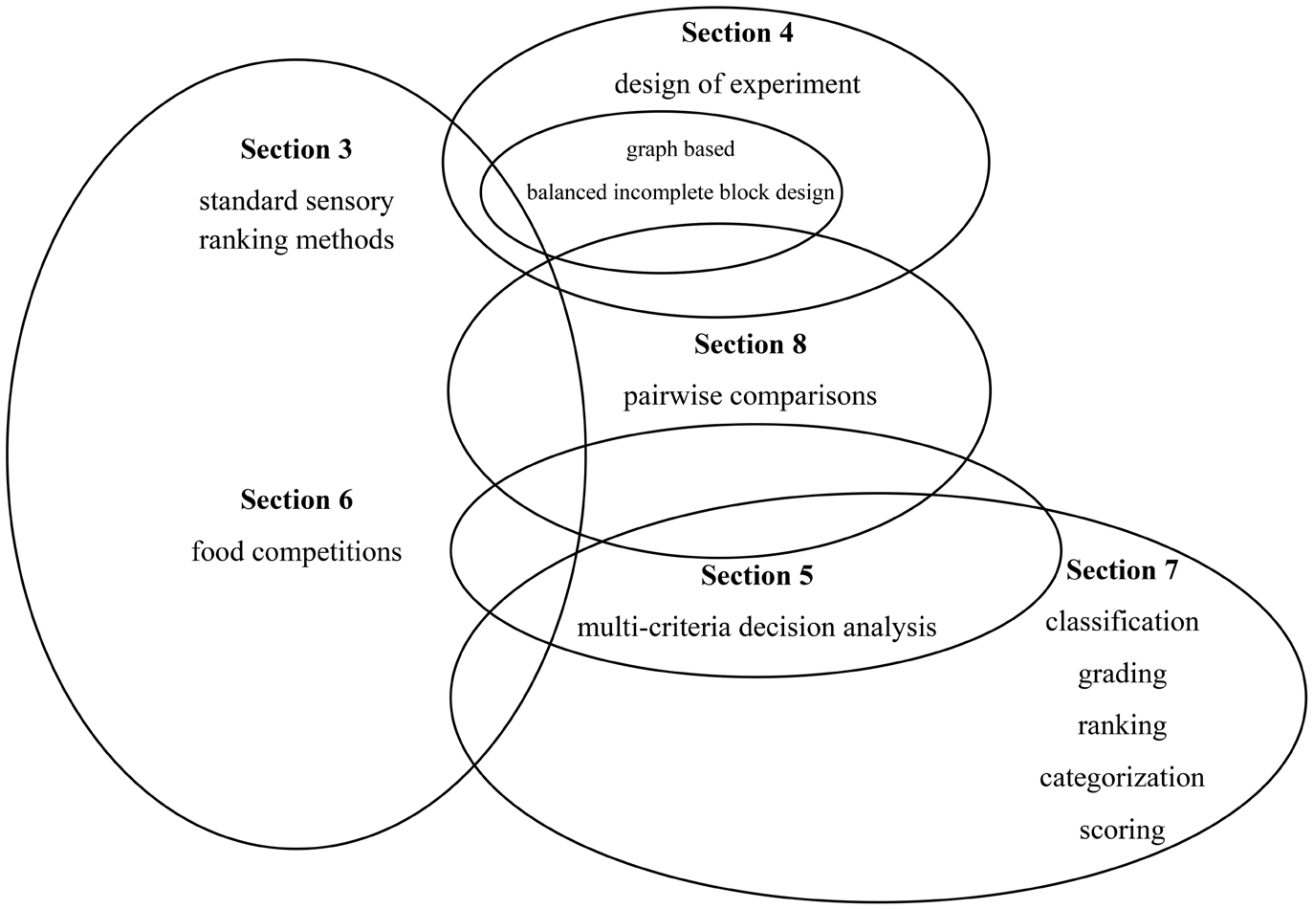
Workflow of the paper.

The paper is organized as follows (Figure 1). Section 2 presents the research methodology. Section 3 focuses on ranking methods in sensory science. Section 4 reviews the design of experiments, including balanced incomplete designs. Section 5 presents the multi‐criteria decision analysis (MCDA) methods, including decision problems and the Analytic Hierarchy Process. International scoring sensory competitions are discussed in Section 6. Different types of classification, grading, ranking, categorizing, and scoring procedures are considered in Section 7. Ranking methods based on paired comparisons and their presence in several disciplines are presented in Section 8. Section 9 synthesizes the trends and future challenges of the previous sections. The methods discussed in Sections 4, 5, 7 and 8 are general methods used in several disciplines besides standard sensory methods and food competitions. However, the organization principle is that the main methodologies of standard sensory ranking methods and food competitions are covered by our overview. Finally, Section 10 concludes.

## 2 Research Methodology

Our research was based on international standard perceptual methods and competitions with a practical focus, and we investigated which comparison‐based questions used in these methods lead to decision‐theoretic models. The identified models were used to provide new perspectives, methodological additions, and solutions for the evaluation of existing standard sensory methods and methodologically related sensory competitions.

The literature search was based on the ISO standards and sensory competition methodologies, and their specific keywords were used to conduct separate searches in scientific databases. Since many of the methods are based on ranking and pairwise comparisons, the decision‐theoretic background of these methods was also reviewed.

Departing from real‐world, practical, standard sensory methods and competitions, our literature review was conducted based on studies dealing with the trends and directions of preference elicitation and assessment in food science. The sources of the published data are academic journals from the main electronic databases (ScienceDirect, Scopus, SpringerLink, Web of Science, Wiley Online Library), as well as PhD dissertations and chapters of academic books. The keywords used in the research were “ranking methods,” “single ranking,” “pairwise ranking,” “pairwise comparisons,” “multi‐criteria ranking,” “food product development,” “food competitions,” “wine competiton,” “extra virgin olive oil competition,” “sensory tests,” “sensory standards,” “International Organization for Standardization,” “ISO/TC 34/SC 12,” “design of experiment,” “balanced incomplete block design,” “Analytic Hierarchy Process,” “multi‐criteria decision making.” Our work has been based primarily on the latest research results, but we have also integrated some previous research findings because of their importance.

In addition to discussing standard sensory methods and competitions, we explore related decision‐theoretical issues and raise the possibility of integrating them with complementary methods from other disciplines.

## 3 Ranking Methods in Sensory Science

### 3.1 Standard Methods in Sensory Ranking

The literature classifies sensory tests according to the selection and training of assessors, the methodological structure, and the purpose of the test. Assessors can be “sensory assessors,” “selected assessors,” and “expert assessors” according to selection and training criteria. The detailed procedures and methods for the selection and training of “selected” and “expert” assessors are described in ISO 8586, while the monitoring of their performance is described in ISO 11132. Among the sensory assessors, consumers do not need to meet any training criteria, but it is essential that the selected consumer group is representative of the target consumer group being assessed. Representativeness includes different sociodemographic factors (gender, age, place of residence, net income, educational level, etc.), consumption habits of a given product (once a day, several times a day, once a week, several times a week, once a month, several times a month, less often), specific consumer groups (vegan, gluten sensitive, lactose sensitive), value characteristics, cohorts, among others. Different types of tasks require the use of different assessors. The methodological structure distinguishes between ranking, difference analysis, and descriptive methods. The choice of the most appropriate method is primarily determined by the purpose of the study. However, in practice, factors such as the products (number of items, type, characteristics), sensory modalities (visual, olfactory, gustatory, auditory, tactile), the condition characteristics of the assessors (sensitivity, allergy, aversion, fatigue, adaptation, health), the vessels for sensory testing, sensory neutralization, should also be taken into account, as well as the test environment (in‐home, in‐store, laboratory, mobile sensory laboratory, virtual reality) and statistical criteria (number of assessors, probability, significance level, proportion of distinguishers, power, sensitivity, specificity, accuracy, reproducibility, confidence interval, etc.) specifications (ISO 6658 2017; ISO 8586 2023; ISO 11132 2021; ISO 11136 2014, 2020).

According to the International Standard for Sensory Ranking, the purpose of ranking is to rank a given set of samples according to one attribute, multiple attributes, or overall perception. However, the standard requires that if it is necessary to determine the ranking with respect to several attributes, this can only be done in a separate test, with the products recoded and tested in a different order by the assessors. The ranking method is suitable for determining the differences, but not for determining the size of the differences. The ranking method can be used in a variety of tests: to evaluate panelists' performance (training of panelists, setting sensory thresholds for panelists or groups of panelists), to preselect samples when evaluating products, to determine the effect of intensity levels of one or more parameters, or to determine liking in an overall liking test (ISO 8587 2006; Sipos et al. 2021).

In ranking, the assessors are given three or more samples at the same time, but the standard recommends the pairwise comparison method (ISO 5495 2005) for ranking two samples. In ranking, the assessors receive the samples in random order, which are ranked according to their intensity or total intensity of a predefined characteristic. The assessors assign a rank number to a given sample based on its ranking (position) in the ranking. After summing these ranks, statistical tests can be performed to determine whether the samples are significantly different or which has a higher/lower rank. Based on the nature of the rankings, a distinction is made between unknown rankings (liking tests) and known or hypothesized rankings (color recognition test, taste threshold test, water minerality, wine reflectivity, sweetening of sweeteners, storage tests, etc.). Consequently, either a two‐sided or one‐sided hypothesis test is performed. The ranking tests can be compared based on their characteristic parameters: test objective, assessor qualifications, number of assessors, statistical methods (Table 1) (Azami et al. 2018; Belingheri et al. 2015; Condurso et al. 2019; ISO 6658 2017, 2006).

**TABLE 1:** Choice of the parameters of the test based on its aim (source: ISO 8587 2006).

Test aim	Assessors qualification	Number of assessors	Statistical method		
			Comparison to a known order	Order of products unknown (products comparison)	
			(assessors performance)	2 products	> 2 products
Performance assessment of individuals	Selected assessors or expert sensory assessors	Unlimited	Spearman test	Sign test	Friedman test
Performance assessment of a group	Selected assessors or expert sensory assessors	Unlimited	Page test		
Product assessment on a descriptive criterion	Selected assessors or expert sensory assessors	Preferably 12–15			
Product assessment on hedonic preference	Consumers	Minimum 60 per group of consumers type (cell and segment)	—		

Overall, standard sensory ranking tests play an important role in the practices of sensory laboratories. From a sensory point of view, their most important advantages are that they are easy to understand and simple to implement for any type of assessor (naive, trained, expert). They can be applied with some products, and they have a wide range of applications, including prescreening, assessor training, quality control, product development, and consumer preference tests. Ranking tests for a single attribute can be implemented quickly and cost‐effectively. Nonparametric statistical tests for evaluation are available in general statistical software packages, and standards, computational aids, and case studies have been developed to assist in design, implementation, and report writing in sensory practices. However, they have the disadvantage that, due to the nature of ranking methods, they can only be ranked and do not provide information on the relative difference between samples. The evaluation of multiple attributes can only be carried out in separate rank order tests. Rank order evaluation of many attributes is not economical, time consuming, and can lead to a loss of motivation of the assessors due to the monotonicity of the tests. The number of samples that can be included in a ranking test is limited (maximum 6), except for the standard BIB design, where more products can be compared, but only for preference tests.

### 3.2 Assessment Types of Ranking Methods

The ranking methods can be divided into three main methodological classes: simple ranking, ranking on a scale (unstructured, structured, category), and pairwise ranking. In simple ranking, the assessors rank the coded products (up to six) for a given sensory attribute by assigning a ranking number to each product (Aliberti et al. 2025). The steps of the implementation are as follows: coarse ranking (separation), fine ranking (pairwise comparison within a group), concatenation (joining the samples from two groups next to each other). In unstructured scale evaluation, the assessors evaluate a specific sensory attribute of the coded sample by placing a mark on a 90–150 mm long line with descriptive terms at both ends, according to their assessment. The products marked on the scale are assigned rank numbers based on the attribute evaluated. In structured scale assessment, the assessor can place the samples on predefined divisions (structure points) on the scale. More than one sample may be assigned to the same class. The category scale is structured sequentially from the categories of intensity values for a given sensory attribute. The values of the category scale can be represented by numerical values, labels, checkboxes, pictograms, or emoticons. The optimal scale (just‐about‐right, JAR) is characterized by an optimal category at the centre and increasing categories of the trait under consideration towards the two edges. The two endpoints of the bipolar scale are marked by the categories “too weak” and “too intense.” The JAR scales are always odd‐numbered (3‐5‐7‐9‐11), with at least three, but typically five items. Several methods have been developed to evaluate JAR scales, one of the most comprehensive methodological summaries is the “Just‐about‐right (JAR) scales: design, usage, benefits, and risks” ASTM MNL63 standard (ISO 4121 2003; ISO 8587 2006; Palma‐Morales et al. 2024; Rothman and Parker 2009).

The advantage of ranking scales is that several types can be combined with numerical, verbal, and visual elements, optimized for the research questions. A well‐designed ranking scale has the advantage of easy to understand, simple to use, containing an appropriate number of distinctive categories, ensuring that categories and labels are not contradictory, and take the research question the training level, age group, and perceptual abilities of the assessors. The ranking method is less influenced by cultural biases (centroid effect, frequency of extreme values, scale range, etc.) compared to other types of scaling evaluations. In contrast, the disadvantage of ranking scales is that they are ordinal and cannot be used to determine either the magnitude or the causes of differences. A rank position can mask differences in intensity and preference. The ranking scale results cannot be converted to interval scales or ratio scales. Cultural and socialization patterns (left‐to‐right or right‐to‐left increasing scores) may affect the results of tests using standard sensory ranking scales. If the ranking pattern is significant, a pairwise comparison test should be conducted for each sample pair, as required by the standards, to determine which JAR category levels or products differ.

The aim of a pairwise comparison study is to identify differences in intensities or preferences by ranking two products. The pairwise comparison test is a forced‐choice test between two alternatives (2‐AFC test). The test can be applied to the difference/similarity of sensory attribute intensity (one‐sided hypothesis testing) or to the difference/similarity of preference (two‐sided hypothesis testing). The method can be applied if the samples have one or more sensory differences, or if one of the samples is a control, gold standard, or benchmark product. Before the test can be carried out, the values that influence the statistical sensitivity of the test (significance level *α* (probability of Type I error), probability *β* of Type II error, proportion of distinguishers *p*_*d*_) must be determined, so that the number of assessors can be established. To achieve the same statistical accuracy, the number of assessors needed for similarity testing should be twice as high as for difference testing. The higher the number of assessors, the more likely it is that small differences will be significant. In practice, the theoretical number of assessors may be limited by several factors—time available for the experiment, number of assessors available, volume of products, etc.—or by using repeated assessments instead of independent assessments to achieve a sufficient number of overall assessments. The evaluation of repeated measurements is not standardized. Assessors must be well informed about the mechanism of assessment, be able to recognize a given sensory attribute and be of the same level of qualification. In the sensory task, assessors are presented with pairs of samples, either monadically or by comparative presentation, where they are asked to evaluate the two samples in a fixed order and then to mark the more intense, preferred sample. After their choice, in the comments section, the assessors can also indicate the reason for the difference, or if they only guessed. Due to the forced‐choice fashion of the method, the assessors must make a choice even if they have only guessed, but a “no difference” or “no preference” answer is not allowed. After aggregating the responses, the number of required correct answers taking *α, β* and pd values into account is tabulated, and the implied confidence interval is used to determine whether there is a difference or similarity in intensity/preference between the two products for the given group of panelists' evaluation. In summary, the method can be used to demonstrate either sensory/preference similarity or sensory/preference dissimilarity. In the case of difference tests, the direction of the difference can be determined, but the level of difference cannot, due to the characteristics of the method (ISO 5495 2005).

The advantage of pairwise comparisons is that they are simple, easy to understand, and can be used to identify small sensory differences (product development, assessor selection, training, monitoring). From the point of view of the assessors, it is easy because the test consists of two samples to be tested and just one question to be answered, which is similar to everyday decisions. An additional advantage compared to classical ranking tests is the possibility to make a comment concerning the reasons for the choice. The disadvantage is that the 2‐AFC test is based on forced choice, so no difference, no preference answers are excluded, even if the assessor does not perceive any difference between the two products. The test does not provide proof of the magnitude of the difference and is only suitable for sensory analysis of homogeneous products and not for multivariate analysis.

## 4 The Design of Experiments

In terms of designing experiments, the traditional one factor at a time (OFAT) or one variable at a time (OVAT) method was used first because it is easy to use and understand, but in other performance characteristics it is inferior to the systematic procedure of statistical design of experiments (DOE). The OFAT and OVAT are resource‐intensive, time‐consuming as each product has to be evaluated separately, can lead to unnecessary runs, and result in typically suboptimal solutions unsuitable for exploring interactions (Beg et al. 2019).

Statistical Design of Experiments (DOE) is a procedure for the systematic evaluation of factors affecting the value of a parameter or parameter set by designing, implementing, analyzing, and interpreting tests. The objective of creating an experimental design is twofold: on the one hand, to create the most efficient experimental design by minimizing the number and complexity of experiments, and on the other hand, to extract the most information. To conclude, an experimental design is an optimization problem; the most information should be extracted in the most economical way for the given objective at the lowest cost (time, energy, money, etc.). Statistical design is the laying out of a detailed experimental design before the experiment is carried out. The logic of experimental design is to deliberately change one or more input factors in order to observe the effect of the changes on one or more output response variables. By manipulating several input factors simultaneously, interactions can also be identified. A reasonable effort should be made to create the simplest experimental design possible, with consideration given to replication (measurement/valid), randomization (sampling), and blocking (assessors), used to control for unexpected and unwanted variation. In general, it is advantageous to consider all experimental factors simultaneously, as this allows the interaction of factors to be investigated, increases relative efficiency, and improves the validity of the main effects of some factors (in the absence of interactions). In many cases, the optimal information set has combinatorial properties that can be described by mathematical techniques (Montgomery 2019; Næs et al. 2011).

Experiment design can be used effectively in sensory testing, in choosing between alternatives (Cantillo et al. 2020), in identifying the most important factors influencing the response (screening) (Yu et al. 2018), in response surface modeling (Chu and Resurreccion 2004; Felberg et al. 2010), and in regression modeling (Yu et al. 2018). The most common empirical models fitted to experimental data are linear or quadratic. In many cases, several iterative, adaptive experiments may be necessary. The general seven steps in the design of experiments (DOE) are as follows: setting objectives (1); selecting process variables (2); selecting an experimental design (3); implementing the design (4); checking the consistency of data and experimental assumptions (5); analyzing and interpreting results (6); and using results (7). The choice of experimental design depends on the objectives of the experiment and the number of factors to be investigated, the amount of resources available, and the degree of first‐ and second‐order error (Trutna et al. 2012) (Table 2).

**TABLE 2:** Design selection guidelines (based on Trutna et al. 2012).

Number of factors	Comparative objective	Screening objective	Response surface objective
1	1 factor completely randomized design	—	—
2–4	Randomized block designs: Latin Square (2 factor), Graeco‐Latin Square (3 factor), Hyper‐Graeco‐Latin Square (4 factor) Balanced Incomplete Block (BIB) design	Full or fractional factorial design	Box–Wilson central composite or Box–Behnken design
5 or more	Randomized block design Hyper‐Graeco‐Latin Square (5 factor) Balanced Incomplete Block (BIB) design	Fractional factorial or Plackett‐Burman design	Screen first to reduce number of factors

The simplest experimental design for comparison is a fully randomized design, testing different levels of a factor. In random block designs, one factor is the primary focus along with other confounders. To reduce or eliminate the contribution of confounders to experimental error, homogeneous blocks are created in which the confounders are held constant and the factor of interest is allowed to vary (e.g., in a product development study, five different levels of a food ingredient are tested). Random block designs are used to test multiple factors for comparison purposes, the most common of which in product‐focused sensory testing is the Latin square method for sample allocation, to determine the different testing order of products per reviewer. The Latin square method is based on a square table (3 × 3, 4 × 4, 5 × 5, 6 × 6, 7 × 7, etc.) in which each number occurs exactly once in each row or column. The number of these combinatorial structures increases exponentially. However, up to 7 × 7, all instances are known, including those that are different from each other in a mathematical sense (they cannot be obtained by permuting the numbers or rows/columns). Standard Latin squares are Latin squares whose first row and first column are 1, 2, 3, …, p. The number of standard Latin squares is 3 × 3: 1, 4 × 4: 4, 5 × 5: 56, 6 × 6: 9408, 7 × 7: 1,694,080 (Fisher and Yates 1953).

In the perceptual experiment design, the assessors are the blocks, and the effect of eliminating the sequence effect and carry‐over effect of the patterns is the confounding factor, and all consecutive pairs of patterns are counterbalanced. In the case of repeated testing of products, the reviewer should aim to test the most different sample sequence from the first test. For the design of the sample allocations, a completely random ordering rather than the Latin square method is also appropriate as it ensures that the effects of confounding factors are eliminated. These sample allocations are intended to provide an unbiased estimation of the sensory attributes of the products. In studies focusing on the evaluation of the performance of the assessors, it is preferred to use the same sample order to ensure that all assessors are equally affected by the bias arising from the sequential ordering of the samples. This sample allocation guarantees that the assessor effect and the product × assessor interaction can be estimated without bias (ISO 11132 2021; Montgomery 2019).

In the case of a screening objective, the experimental design aims to select or screen out a few important main effects besides many less important effects. Within these designs, all possible combinations may be tested in a full factorial design, or only a subset of all possible combinations may be tested in a fractional factorial design. The full factorial design is characterized by symmetry (all columns have the same number of “+” and “–” values), normality (the value of the factors in the matrix is always +1 or −1.), orthogonality (the scalar product of any two column vectors of the matrix is equal to zero), and rotation (the accuracy of the determination of the optimisation parameter is equal at equal distances from the experiment and does not depend on the direction, that is, we obtain an equally accurate estimate of the optimum of the experimental design, regardless of the direction from which the optimum is approximated) (Granato and Ares 2014).

The advantage of a full factorial design is that, in addition to the main effect, the effect of all interactions, combinations, and quadratic effects can be examined, but the disadvantage is that the multiplicative relationship between the factors and the factor levels leads to a combinatorial explosion in the number of patterns, which significantly increases the number of products to be compared. Therefore, several methods have been developed to reduce the total number of combinations in fractional/partial factorial designs, in the case where the interactions are negligibly small compared to the main effects. Mixed‐level systems require complex designs. However, the estimates of main effects are not as accurate because the averages are derived from fewer measurements. If the number of factors is five or more, a full factorial design requires a large number of runs and is not very efficient, so a fractional factorial design or a Plackett–Burman design is a better choice (Antony 2023).

**FIGURE 2:**
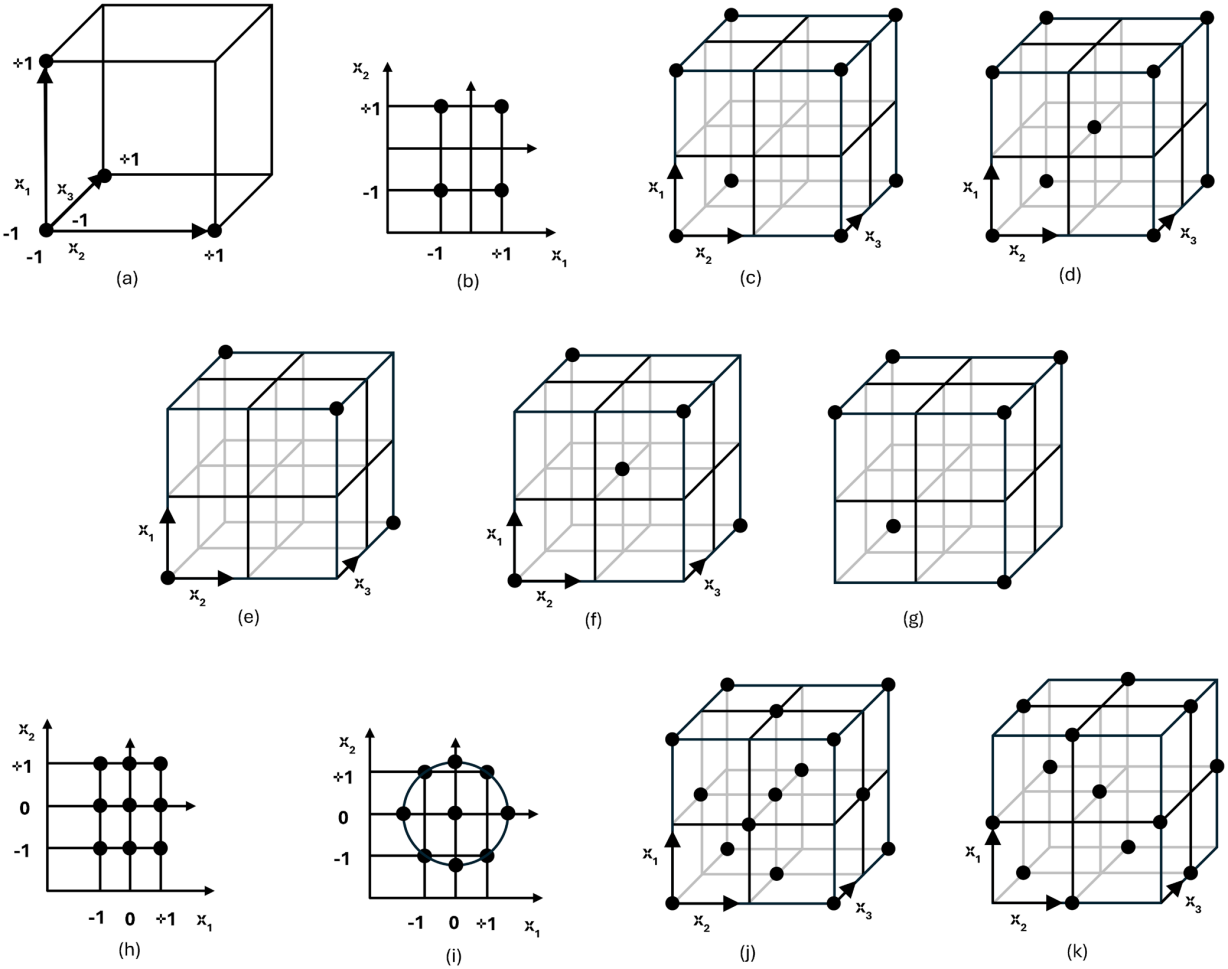
One factor at a time and design of experiments for screening and response surface objectives: (a) one factor at a time, (b) 22 full factorial design, (c) 23 full factorial design, (d) 23 full factorial design with an added center point, (e) 23–1 fractional factorial design with design points as spheres, (f) 23–1 fractional factorial design with added central point, (g) Plackett‐Burman design, (h) Box‐Wilson central composite design: Rectangular domain, (i) Box‐Wilson central composite design: Spherical domain, (j) Box‐Wilson central composite design: Cubic domain, (k) Box–Behnken design for three factors in cubic domain (based on Beg et al. 2019; Montgomery 2019; Trutna et al. 2012).

For the purpose of the response surface method (RSM), the experimental design allows the estimation of the main effects as well as the interactions and their spillover effects, so that the response surface under investigation can be plotted. The Box–Wilson central composite design, which consists of an embedded factorial or fractional factorial design with centers, supplemented by a set of “star points” that allow the curvature to be estimated. The Box–Behnken plan, on the other hand, is an independently rotatable quadratic plan, with treatment combinations at the center and at the center of the edges of the process space. Each factor requires three levels, typically fewer treatment combinations and fewer runs than the Box–Wilson plan. It is important to point out that both the Box–Wilson central composite plan and the Box–Behnken plan contain regions of poor predictive quality (Beg et al. 2019; Selvamuthu and Das 2018) (Figure 2).

Expert sensory profiling and consumer testing are typically carried out in a full block design, where each product is tested in each block by the assessors. However, it is a common feature of product development tasks that consumer tests need to be performed on many products (*n* > 6) to be tested at the same time. The use of excessive sample numbers causes sensory fatigue, excessive mental workload, and loss of assessor motivation, which can significantly impact test reliability. To solve this problem, the balanced incomplete block design (BIBD) was developed, in which assessors evaluate only a specified subset of sample combinations in a block. The BIBD is designed so that all samples are assessed by the same number of assessors and all possible sample pairings are assessed by the same number of assessors in a block (Cochran and Cox 1957; ISO 29842 2011, 2015; Ramírez‐Jiménez et al. 2018; Zheng et al. 2023).

**FIGURE 3:**
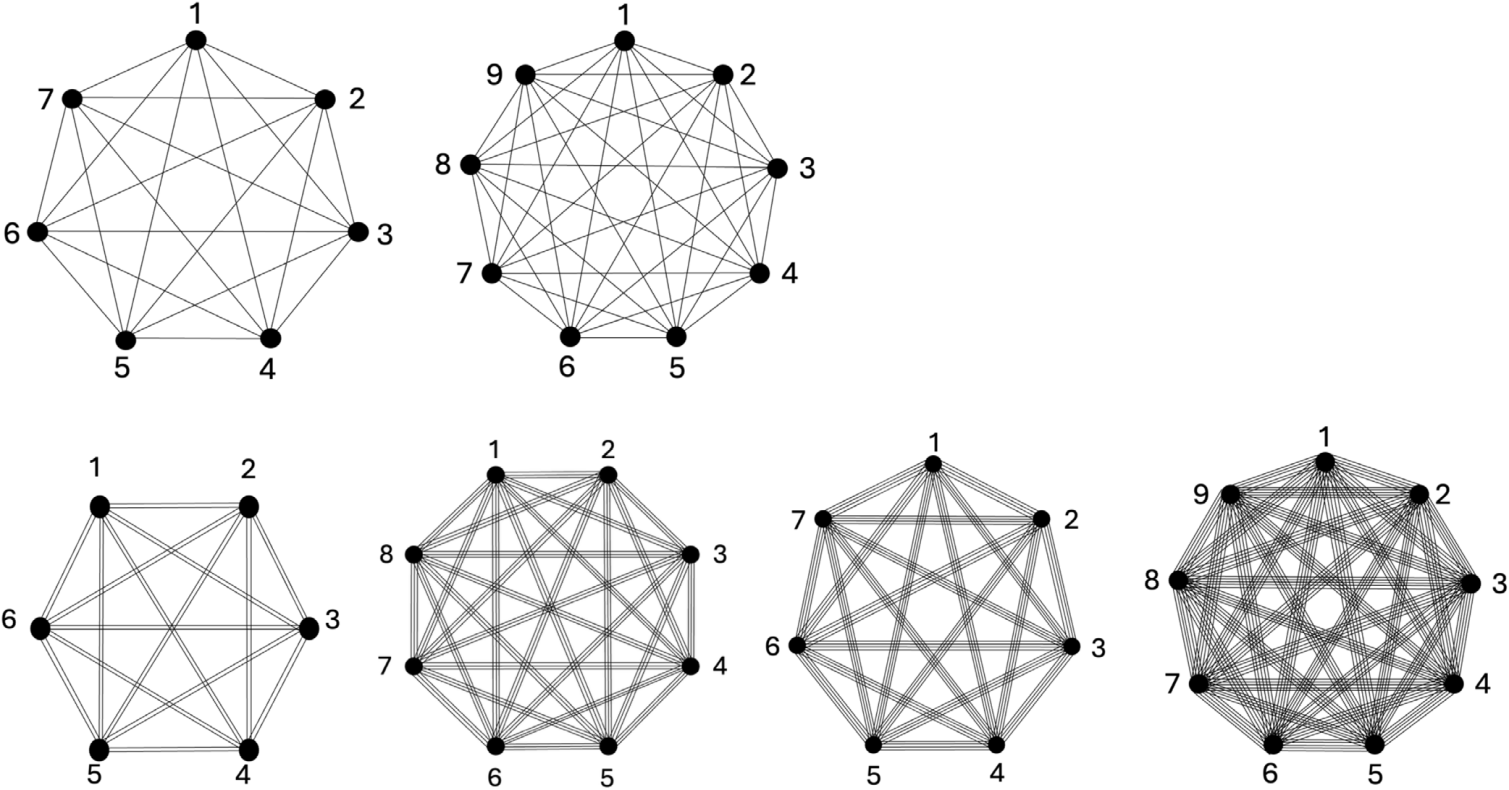
Single complete BIBD graphs (7 samples, 9 samples) and double (6 samples), triple (8 samples), quadruple (7 samples), quintuple (9 samples) multi complete BIBD graphs (based on ISO 29842 2011, 2015).

The international standard proposes different BIB designs, defined by the following parameters: number of test samples (*t*), number of samples evaluated by an assessor in a single session (*k*), total number of blocks/assessors in one repetition of the BIB design (*b*), number of times each test sample is evaluated in one repetition of the BIB design (*r*), number of times each pair of samples is evaluated by the same number (*λ*) of assessors, and number of times (*p*) the basic BIB design is repeated. The BIB designs, given in assignment table format, can also be represented by graphs, with vertices associated with the sensory test samples and edges corresponding to the comparisons between pairs of samples. The final result of the BIBD is a complete or multiple complete graph (multigraph) (Figure 3).

In a BIBD‐based sensory test, an assessor evaluates several products, which can be pairs (2), triads (3), tetrads (4), pentads (5), or sextads (6). The standard provides detailed assignments for each BIBD, specifying which assessor tests which samples. The choice of the most appropriate BIBD for the purpose can be influenced by the degree (*d*), the number of edges of the BIBD (*e*), and the total number of sensory samples to be tested (*s*), in addition to the parameters specified in the standard (*t*, *k*, *b*, *r*, *λ*). The *λ*‐multigraph is regular; the degree of each vertex is *d*.

However, it is not possible to select an optimal level of parameter combinations in all respects, since the parameter pairs to be minimized (*t*, *k*, *b*, *s*) and maximized (*r*, *λ*, *d*, *e*) are not independent of each other. Furthermore, the number of samples, the nature of samples, and the number of assessors are the most common determinants in sensory testing practice (Table 3).

**TABLE 3:** Comparison of BIB designs with standard and graph‐theoretic parameters (based on ISO 29842 2011, 2015).

	Standard BIB design parameters							Graph theoretical parameters	
		Number of test samples (number of columns in the table format) (number of vertices in the graph)	Number of samples evaluated by an assessor in a single session (number of “X's” in any row of table format)	Total number of blocks/assessors in one repetition of the BIB design (number of rows in the table format)	Number of times each test sample is evaluated in one repetition of the BIB design (number of “X's” in any column of table format)	Number of assessors evaluating each pair of samples (how many times the complete graph is multiplied)	Total number of sensory samples (portion) to be prepared for testing (total number of “X's” in the table format)	Degree of vertices	Number of edges
		*t*	*k*	*b*	*r*	*λ*	*s*	*d*	*e*
		*t*	*k*	*b*	*r* = *k* × *b*/*t*	*λ* = *b* × *k*/[t × (*t*−1)/2]	*s* = *k* × *b*	*d* = *λ* × (*t*−1)	*e* = *λ* × *t* × (*t*−1)/2
a	All possible pairs of 3 samples	3	2	3	2	1	6	2	3
b	All possible pairs of 4 samples	4	2	6	3	1	12	3	6
c	All possible triads of 4 samples	4	3	4	3	2	12	6	12
d	All possible pairs of 5 samples	5	2	10	4	1	20	4	10
e	All possible triads of 5 samples	5	3	10	6	3	30	12	30
f	All possible tetrads of 5 samples	5	4	5	4	3	20	12	30
g	All possible pairs of 6 samples	6	2	15	5	1	30	5	15
h	All possible triads of 6 samples	6	3	10	5	2	30	10	30
i	All possible tetrads of 6 samples	6	4	15	10	6	60	30	90
j	All possible pentads of 6 samples	6	5	6	5	4	30	20	60
k	All possible pairs of 7 samples	7	2	21	6	1	42	6	21
l	All possible triads of 7 samples	7	3	7	3	1	21	6	21
m	All possible tetrads of 7 samples	7	4	14	8	4	56	24	84
n	All possible pentads of 7 samples	7	5	21	15	10	105	60	210
o	All possible sextads of 7 samples	7	6	7	6	5	42	30	105
p	All possible pairs of 8 samples	8	2	28	7	1	56	7	28
q	All possible triads of 8 samples	8	3	56	21	6	168	42	168
r	All possible tetrads of 8 samples	8	4	14	7	3	56	21	84
s	All possible pentads of 8 samples	8	5	56	35	20	280	140	560
t	All possible hexads of 8 samples	8	6	28	21	15	168	105	420
u	All possible pairs of 9 samples	9	2	36	8	1	72	8	36
v	All possible triads of 9 samples	9	3	12	4	1	36	8	36
w	All possible tetrads of 9 samples	9	4	18	8	3	72	24	108
x	All possible pentads of 9 samples	9	5	18	10	5	90	40	180
y	All possible sextads of 9 samples	9	6	12^a^	8^a^	5	72	40	180
z	All possible pairs of 10 samples	10	2	45	9	1	90	9	45
aa	All possible triads of 10 samples	10	3	30	9	2	90	18	90
bb	All possible tetrads of 10 samples	10	4	15	6	2	60	18	90
cc	All possible pentads of 10 samples	10	5	18	9	4	90	36	180
dd	All possible sextads of 10 samples	10	6	15	9	5	90	45	225

^a^ Corrected in ISO 29842 (2015).

**FIGURE 4:**
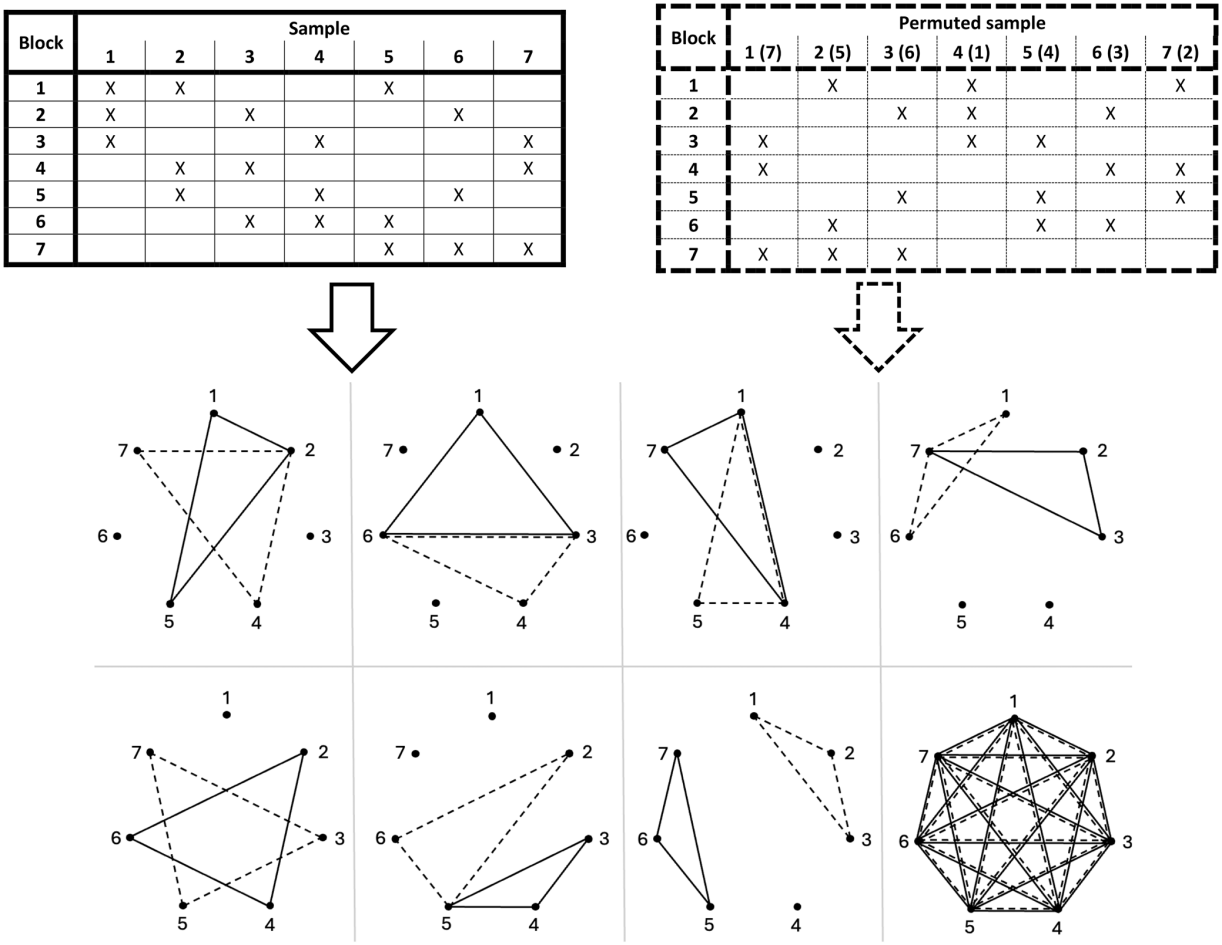
Two mechanisms of complete BIBD graph with all possible pairs: Seven triads based on standard (solid line) (ISO 29842 2011, 2015), seven triads on a permutation of relabelled vertices (dashed line).

At the same time, it may be necessary to increase the reliability of the results, typically by increasing the number of samples and assessors, preferably by repeating each block p times, that is, by multiplying the entire graph. One possible solution to this is to implement the test (full graph) with the same sample allocation but with new assessors. Another possible solution is to test the products by involving new assessors but with a different sample assignment, for example, by permutation and relabeling the vertices (Figure 4).

For the required level of precision, the BIB design needs to be repeated several times. As a standard, for quantitative scale scoring, a modified ANOVA and Fisher's LSD post hoc test depending on the design are required, while for ordinal scale scoring, Friedman's test and Fisher's LSD post hoc test are required. The appendix provides several BIBDs, which are characterized by the properties that all products occur equally often in a given position (balanced order) and all pairs of a given order are included equally often (minimalization of the carry over effect). Software on several platforms for BIBD generation is available, and the evaluation is used in practice (R‐project, JMP, SPSS, SAS) (Gacula et al. 2009; ISO 29842 2011, 2015).

The advantage of the design of experiment is that more information can be obtained from fewer tests than with univariate methods (OFAT/OVAT). It also allows the simultaneous investigation of several sensory properties and their interactions. It requires less testing time and fewer samples than comparing all samples. Response surface methods (e.g., Box–Behnken, Box–Wilson) allow the mapping of curves. Latin square and BIBD methods help to reduce bias. BIBD reduces sensory fatigue for assessors if large sample sizes are involved, allows all samples to be evaluated at the same frequency, and is supported by software. The disadvantage of DOE is that its design and the evaluation of the results require statistical knowledge and expertise, and the creation of the experimental design is time‐consuming. Replication is needed to increase the accuracy of BIBD.

## 5 Multi‐Criteria Decision Analysis

The solution of the majority of decision problems requires the consideration of several, often conflicting criteria. For example, in choosing an optimal investment portfolio, at least the expected returns and risks should be taken into account. Furthermore, usually there does not exist a perfect alternative that is ideal with respect to all criteria—in the previous example, the stock of no company can offer the highest return with the minimal level of risk. Therefore, a compromise solution should be found.

The first idea to address a multi‐criteria decision problem is using the weighted sum: a weight is associated with each criterion and the overall performance value of an alternative is derived by aggregating its performances with respect to all criteria according to these weights. However, this naïve method can be applied only if all data are expressed in exactly the same unit, the preferences are linear, the criteria are independent, etc. In other words, such a simple approach may easily lead to insensible outputs.

Consequently, since the 1970s, several multi‐criteria decision analysis (MCDA) methods have been developed to support decision‐makers facing decision problems involving multiple criteria. They have been successfully applied to solve decision‐making problems in several areas, including climate change, commerce, energy, healthcare, social policy (Basílio et al. 2022), and even food market segmentation (Casas‐Rosal et al. 2023).

Roy (1981) has identified four basic types of decision problems:

1. Choice problem where the goal is selecting the best alternative or to find a subset of alternatives that are incomparable due to the imprecision of data or can be optimal under reasonable preferences. A classical application is filtering each dominated alternative, which is dominated by another alternative with respect to all relevant criteria.
2. Sorting problems where the options are classified into different categories with an intrinsic definition. For example, foods can be divided into three to five groups according to their quality attributes such as color, odor, and sweetness.
3. Ordering problem where the aim is to rank the alternatives in a decreasing preference order from the best to the worst.
4. Description problem where the options and their consequences are described to understand the main characteristics of the decision problem. This formulation is often included in the three preceding ones.

The most popular methods to solve multi‐criteria decision problems can be found in Ishizaka and Nemery (2013, table 1.2). They usually have a supporting software package, too (Ishizaka and Nemery 2013, table 1.3). These methods are compared in Majumder and Salomon (2024, table 1) with respect to the required inputs and mathematical models and extensively discussed in Ishizaka and Nemery (2013).

The Analytic Hierarchy Process (AHP), developed by Thomas L. Saaty (1977, 1980), is one of the most popular MCDA methods. It consists of the following steps:

– Structuring the problem as a hierarchy by formulating the goal, the alternatives to be evaluated, and the criteria used to assess the alternatives;
– Establishing priorities at each level of the hierarchy based on pairwise comparisons of the elements;
– Synthesizing these judgments to determine the overall priorities with respect to the decision goal;
– Checking the consistency of the pairwise comparisons;
– Sensitivity analysis.

Although the last two steps are optional, they could be important to confirm the robustness of the results.

The AHP methodology does not require the construction of an explicit utility function. There is no need for numerical judgments; a relative verbal appreciation may be sufficient. However, the translation of these verbal answers to numerical values is far from being straightforward (Cavallo and Ishizaka 2023).

Another advantage of AHP resides in using pairwise comparisons: the decision maker can focus on comparing only two criteria/alternatives at the same time. Nonetheless, this simplification has some price. First, the pairwise comparisons might be *inconsistent*; for instance, if alternative A is two times better than alternative B, and alternative B is three times better than alternative C, it is not guaranteed that alternative A will be judged six times better than alternative C by the decision maker. This calls for checking consistency, similar to sensory testing (Sipos et al. 2025). Second, the number of pairwise comparisons needed is a quadratic function of the number of alternatives, which might be excessive if the latter is relatively high (e.g., 10 alternatives require 45 pairwise comparisons). AHP can be extended by allowing for missing pairwise comparisons (Bozóki et al. 2010), analogous to incomplete block designs.

Finally, the last step of the AHP is usually a sensitivity analysis, which examines the impact of slight changes in the input data. It generates different scenarios that are still reasonable due to the uncertainty of the preferences of the decision‐maker or the inaccurate characteristics of the alternatives. If the ranking is largely insensitive to these modifications of the input, the result of the model is said to be robust. The sensitivity analysis also helps identify the important variables that significantly affect the final decision.

The advantage of MCDA methods resides in the assessment of several attributes in the same framework. In AHP, the assessor makes pairwise comparisons where verbal assessment is sufficient. However, as the number of alternatives increases, the number of comparisons increases quadratically, which can cause sensory fatigue. Consistency checks may be necessary due to possible inconsistencies. Sensitivity analysis could be time‐consuming but is inevitable to examine the robustness of the results. The quantification of the verbal evaluation is not straightforward, and subjective weighting of different sensory attributes may also influence the decision.

## 6 International Scoring Sensory Competitions

Several food competitions exist around the world, where the products are specifically assessed for their sensory qualities. A large number of local, regional, national, and international food competitions are organized, with the main product categories being pleasure food (wine, beer, whiskey, champagne, coffee, chocolate, cheese, olive oil, mineral water, etc.). Food competitions date back to the past, but since the 2000's their number has been growing dynamically (Bitter 2017; Honoré‐Chedozeau et al. 2015; Rébufa et al. 2021).

The goals of creating food competitions are complex:

– On the product side, to rank the products entered, classify them into categories and quality classes (Hodgson and Cao 2014; Malfeito‐Ferreira et al. 2019; Rébufa et al. 2021);
– On the product producer side, gaining market advantage, increasing market share, increasing reputation by communicating the achievement, improving knowledge of local/regional/national/international market products/product developments/new technology results, feedback on product perception (Cao 2014; Girard et al. 2023; Malfeito‐Ferreira et al. 2019; O'Sullivan et al. 2011; Parr et al. 2006; Rébufa et al. 2021; Štefan et al. 2020);
– On the product category side, enhancing image in comparison with other product categories (Girard et al. 2023; Rébufa et al. 2021);
– On the consumer side, to help decision‐making and choice (Cao 2014; Girard et al. 2023; Honoré‐Chedozeau et al. 2015; O'Sullivan et al. 2011; Rébufa et al. 2021);
– Competitions themselves compete in order to generate profits, gain greater recognition, prestige, and achieve a better market position than other competitors (Cliff and King 1996; Girard et al. 2023).

The geographical location, regulations, and events of food competitions show a high degree of heterogeneity in several aspects: product/product type (Malfeito‐Ferreira et al. 2019; Rébufa et al. 2021), number of products to be tested (ISO 6658 2017), knowledge of assessors/judges consumers, trained assessors, experts, food sector experts (Hopfer and Heymann 2014; Rébufa et al. 2021; Scaman et al. 2001), training (Hopfer and Heymann 2014; Scaman et al. 2001), proficiency (Hopfer and Heymann 2014; Scaman et al. 2001), sensory attributes to be evaluated (Girard et al. 2023; Hopfer and Heymann 2014; Malfeito‐Ferreira et al. 2019; Rébufa et al. 2021), testing conditions (Hopfer and Heymann 2014), aggregation rules of the scores (Bodington and Malfeito‐Ferreira 2019; Cicchetti 2009; Cliff and King 1999; Parr et al. 2006; Rébufa et al. 2021), prizes/award system (Bodington and Malfeito‐Ferreira 2019; Cicchetti 2009; Malfeito‐Ferreira et al. 2019; Parr et al. 2006; Rébufa et al. 2021), communication conditions of prizes (Rébufa et al. 2021), competition awareness and recognition (Rébufa et al. 2021). The results of food competitions are usually reached by considering several aspects together, so overall, it is a multi‐criteria decision‐making task where different alternatives and different combinations of products have to be evaluated according to the given competition rules. Food competitions almost exclusively use scoring evaluation methods and have food‐specific rules. Individual subjectivity is unavoidable as judges have to answer questions on preference in addition to questions on intensities. Sensory attributes are evaluated by rating and scoring each product and using this information to formulate the final results. The challenge of food competitions is how to transform the often subjective quality into objective‐looking numbers, rankings, results, and prizes. As a result, objective and subjective evaluation criteria are mixed in the final results (Parr et al. 2006).

All food competitions require a reliable, scientific, product‐independent scoring system. One solution to this problem is the “Sense‐Award” scoring system created by sensory experts from the University College Cork (UCC) and University of Copenhagen (UCPH). The products are evaluated in two stages. The first stage is judged by consumers who are familiar with the product category. In the second stage, the top five products from the first stage are judged by assessors from different parts of the food industry: trade buyers, chefs, retailers, food writers, and again, one consumer from each product category. The products are assessed in a blind test, using a simple scoring system (reject = 0, fair = 1–2, good = 3–4, very good = 5–6, excellent = 7–8, gold standard = 9–10) in six separate categories (appearance, aroma–taste, stock, overall quality, likability [consumer relevance]). Gold, silver, and bronze awards are given to several products in each product category (O'Sullivan et al. 2011).

It can be concluded that food competitions involve a number of subjective elements, product comparison problems and sensory anomalies: assessors/judges with different skills, qualifications, backgrounds (Bitter 2017; Hopfer and Heymann 2014; Hodgson 2008; Scaman et al. 2001), assessors/judges with different performance (Berg et al. 2022; Bitter 2017; Cao and Stokes 2010; Cliff and King 1996, 1997; Scaman et al. 2001), lack of prescreening/performance evaluation (Bodington 2020; Cliff and King 1996; Hodgson and Cao 2014; Hopfer and Heymann 2014; Scaman et al. 2001), varying product testing conditions (product temperature, light environment, air quality) (ISO 8589 2007), sensory fatigue, mental load, loss of motivation from excessive sample size (ISO 6658 2017), inappropriate sample coding (ISO 6658 2017), sample order bias (Cao 2014; Cliff and King 1996; Honoré‐Chedozeau et al. 2015; Hopfer and Heymann 2014), ambiguous scoring systems (Balinski and Laraki 2013; Bitter 2017; Hodgson 2009), scoring systems with specificities (attribute definition, attribute weighting, attribute substitutability) (Cliff and King 1999; Hopfer and Heymann 2014; Malfeito‐Ferreira et al. 2019; Rébufa et al. 2021; Wright 2021), problems arising from incomplete matrix comparisons (Cicchetti 2009; Hodgson 2009), inconsistent judgments (judges' perception and judgment, unclear rules, heterogeneous testing conditions) (Balinski and Laraki 2013; Bitter 2017; Cao 2014; Cliff and King 1996; Hodgson 2008, 2009; Hodgson and Cao 2014; Honoré‐Chedozeau et al. 2015; Scaman et al. 2001; Stuen et al. 2015). These problems and nonconformities could be mostly eliminated by integrating the sensory standards developed and continuously improved by “ISO/TC 34/SC 12 Sensory analysis,” thus, the planning, implementation and evaluation of sensory competitions could certainly provide more reliable results.

Food competitions aggregate several criteria with an associated weighting scheme and typically involve a large number of assessors. However, the sensitivity of the results to the weighting scheme, the reliability of the assessments/judgments and the consistency of the assessors are usually not examined, since the aggregation of a large number of responses is assumed to compensate for possible errors. Competitions and their rules (criteria, weights, ranking/scoring, and aggregation) vary from product to product, and there are different rules even for the same product. Hence, the ranking of the same set of products depends on the rules of the competition, even if the scorers are the same.

Two traditional product examples, wine and olive oil competitions are discussed in detail. Wine competitions are of particular interest in sensory science. One of the most prestigious competitions is organized by the OIV (International Organisation of Vine and Wine 2009). The multi‐criteria evaluation system of OIV competition rules is given in Table 4. It is formally a 100 points scoreboard; however, even the worst possible wine receives 40 points, implying a range of 60 points. Experts evaluate the wines with respect to professional criteria, while the last criterion (general impression) measures overall evaluation. The criteria weights are reflected by the scores. A hypothetical example is denoted by bold; the wine receives 5 + 8 + 5 + 6 + 14 + 5 + 7 + 6 + 19 + 10 = 85 points (Table 4). Note that the scores of 'positive intensity' (nose and taste), denoted by italic, are not distributed evenly.

**TABLE 4:** The multi‐criteria evaluation system of OIV competition rules of still wines (based on International Organisation of Vine and Wine 2009). A hypothetical example are denoted by bold. The unevenly distributed scores of 'positive intensity' (nose and taste) are denoted by italic.

Criteria		Evaluation categories/scores					Implicit weight (max–min)
		Excellent	Very good	Good	Fairly good	Bad	
		Very strong	Strong	Average	Light	Very light	
		Excellent	Limpid	Ambiguous	Moderate	Very cloudy	
Visual	Limpidity	**5**	4	3	2	1	4
	Aspect other than limpidity	10	**8**	6	4	2	8
Nose	Genuineness	6	**5**	4	3	2	4
	Positive intensity	*8*	*7*	***6***	*4*	*2*	*6*
	Quality	16	**14**	12	10	8	8
Taste	Genuineness	6	**5**	4	3	2	4
	Positive intensity	*8*	***7***	*6*	*4*	*2*	*6*
	Harmonious persistence	8	7	**6**	5	4	4
	Quality	22	**19**	16	13	10	12
Harmony	Overall judgment	11	**10**	9	8	7	4
	Total	100	86	72	56	40	

*Note:* A hypothetical example are denoted by bold. The unevenly distributed scores of ‘positive intensity’ (nose and taste) are denoted by italic.

An equivalent system is presented in Table 5. The weights of criteria are now given explicitly, and the maximal/minimal score with respect to each criterion is 4/0. The total score is calculated as 40 + the weighted sum of scores. Both systems result in identical total scores for the same scoreboard. In our example, 40 + 1 × 4 + 2 × 3 + 1 × 3 + 1.5 × 2.667 + 2 × 3 + 1 × 3 + 1.5 × 3.33 + 1 × 2 + 3 × 3 + 1 × 3 = 40 + 45 = 85, the same as before (Table 5). The transformed scores of the hypothetical example of Table 4 are denoted by bold in Table 5 as well. As before the transformed scores of 'positive intensity' (nose and taste), denoted by italic, are not distributed evenly.

**TABLE 5:** An equivalent system with explicit weights of criteria and identical (max = 4, min = 0) values (based on International Organisation of Vine and Wine 2009). The transformed scores of the hypothetical example are denoted by bold. The unevenly distributed transformed scores of 'positive intensity' (nose and taste) are denoted by italic.

Criteria		Evaluation categories/scores (max = 4, min = 0)					Explicit weight	Constant added
		Excellent	Very good	Good	Fairly good	Bad		
		Very strong	Strong	Average	Light	Very light		
		Excellent	Limpid	Ambiguous	Moderate	Very cloudy		
Visual	Limpidity	**4**	3	2	1	0	1	1
	Aspect other than limpidity	4	**3**	2	1	0	2	2
Nose	Genuineness	4	**3**	2	1	0	1	2
	Positive intensity	*4*	*3.333*	***2.667***	*1.333*	*0*	1.5	2
	Quality	4	**3**	2	1	0	2	8
Taste	Genuineness	4	**3**	2	1	0	1	2
	Positive intensity	*4*	***3.333***	*2.667*	*1.333*	*0*	1.5	2
	Harmonious persistence	4	3	**2**	1	0	1	4
	Quality	4	**3**	2	1	0	3	10
Harmony	Overall judgment	4	**3**	2	1	0	1	7

*Note:*The transformed scores of the hypothetical example are denoted by bold. The unevenly distributed transformed scores of ‘positive intensity’ (nose and taste) are denoted by italic.

However, it is far from clear whether the evaluators focus on the verbal descriptions (excellent, very good etc.) or the corresponding scores. Note that the results of such experts' evaluation can differ from consumers' choice, which is not necessarily based on the same criteria (and their weights of importance).

Extra virgin olive oil (EVOO) is one of the most important ingredients of the Mediterranean diet. Therefore, a number of international competitions exist for EVOOs (Rébufa et al. 2021). Oil producers are interested in participating in these competitions as a useful marketing tool. Judges award prizes to the oils to recognize the work of their produces and guide consumers. Perhaps the most prominent competition is the IOC Mario Solinas, organized by the International Olive Council (IOC). The 2023 edition has been contested by 117 olive oils, more than 80% of them from three countries: Portugal (16), Spain (63), and Tunisia (19) (IOC 2023b). In this competition, olive oils are evaluated using a scoring system with a maximum of 100 points: 35 points for olfactory sensations, 45 points for gustatory‐retronasal sensations, and 20 points for final olfactory gustative sensations. Other competitions use different scoring systems; for example, EVOOLEUM gives 50, 40, and 10 points for these criteria, respectively. Usually, the competitions award medals (gold, silver, bronze) to the olive oils achieving the highest scores in each category.

There is also an intercompetition classification system called EVOO World Ranking (see http://www.evooworldranking.org). It is a nonprofit initiative aiming to promote the world's most awarded EVOO's worldwide to the consumers. Currently, it takes the results of 38 contests into account: 25 from Europe, 6 from Asia, 4 from Latin America, and 5 from North America. The contests are divided into categories by importance in the world and area, number of samples, number of countries, and impact in major consuming areas of the world. Every contest has a score of 5, 6, 6.5, 7, 8, 9, or 10, but 10 is given only to the IOC Mario Solinas. The awards collected in these competitions are also transformed into scores; the first place means 8 points, the second 6.5, and the third 5, with additional points for special prizes. For each olive oil, the scores of contests and the prizes are multiplied and added to get the total score for the year.

Competition rules differ not only between product types, but often also between countries, regions and competitions, making it difficult to compare results. It is almost impossible to judge the true value of the prizes won in food competitions, but more objective, transparent and fair competitions help both the producer and the consumer. The credibility of the results would be significantly improved by applying the standard rules uniformly and making the assessments and data available ex post. It would be necessary to establish uniform rules, uniform test conditions, uniform level of assessor qualifications and skills, and a uniform test protocol for each product category. Comparison of the methods and assessment procedures used in competitions and the identification of noncompliances have not been fully carried out to date. A collection of scoring sensory competitions is supplemented (Data S1).

The advantage of sensory competitions is that the products are evaluated in a complex, multidimensional way by independent experts or a jury. The prizes and placings awarded can improve the image and prestige of the product and the product category, help consumer decision‐making, and can be used effectively for marketing and advertising purposes. Sensory competitions provide an opportunity to compare a product to its competitors and help to build professional relationships. However, the perception, training, and experience of the assessors may vary, as may the rules and evaluation criteria of competitions, which can influence the results. Brand awareness and visibility may affect the scoring of the jury and hence the placings achieved. The costs of participation (entry fees, sending samples, travel, promotion, etc.) can be prohibitive, especially for smaller companies. A further problem is that the scores obtained in the competitions do not necessarily reflect the real differences between products, and the scoring principles are often unavailable, which can reduce the credibility and transparency of the competitions.

## 7 Classification, Grading, Ranking, Categorizing, and Scoring Procedures

In food testing, there is a difference between classification, grading, ranking, categorizing, and scoring. In classification, the results obtained for a product type can be summarized according to the frequency of each category. Then, using the chi‐square test, one can tell how two or more types of products are distributed between the different categories. The null hypothesis that the distributions are the same is tested against the alternative hypothesis that they are different. In the case of grading, the data can be summarized for classification purposes. The results can be summarized in terms of medians. The products can be statistically compared with rank sum tests, but some modifications may be necessary due to the large number of tied ranks. If the data obtained for a sample are used as a basis for making a decision on a large quantity (a “batch”), the characteristics of the appropriate sampling design are obtained from ISO 2859 (all parts) and ISO 3951. When ranking samples, assessors can perform statistical tests to determine whether the samples are significantly different (rank sum tests). Further tests can be performed to determine whether a particular sample has a significantly higher or lower rank compared to other samples. Categorizing means that the ranks are grouped into homogeneous clusters. In the case of categorization on a noncontinuous scale with few points, the results obtained for a sample can be treated in the same way as for classification. Continuous data or noncontinuous data with a high number of points can be grouped and summarized according to the frequencies associated with each interval. When categorizing more than one sample, the nonparametric method is preferable. When scoring, the results obtained for a sample are summarized as a median or an average (arithmetic mean) with some degree of dispersion (such as range or standard deviation). If there are only two samples and the normal distribution of scores is acceptable, a *t*‐test can be used (see ISO 2854 1976). If the scores are derived from more than two samples, analysis of variance (ANOVA) should be used. If the scores of individual samples do not follow a normal distribution, then the nondistribution (independent of distribution) methods should be used (ISO 2859‐1 1999; ISO 2859‐2 2020; ISO 2859‐3 2005; ISO 2859‐4 2020; ISO 2859‐5 2005; ISO 3951‐1 2022; ISO 3951‐2 2013; ISO 3951‐3 2007; ISO 3951‐4 2011; ISO 3951‐5 2006; ISO 3951‐6 2023; ISO 6658 2017) (Table 6).

**TABLE 6:** Classification, grading, ranking, categorizing, scoring procedures (based on ISO 6658 2017; ISO 4121 2003).

Method	Example
Grading	1st class 2nd class …
Ranking	1st best one 2nd best …
Categorization	For example, excellent–very good–good–fairly good–bad (ISO 4121 2003)
Classification	Verbal labeling for example, white/red/rose (wine) (without assessment)
Scoring	1–2–3‐4‐5

The quantification of preferences is often based on the assumption that the overall utility of products or services can be decomposed to criterion‐wise utilities, being independent of each other. Total scores not only induce a ranking but also indicate the overall performance compared to an ideal, perfect alternative. Total scores also quantify the differences between alternatives. Scoresheets are typical examples when the evaluators are able to assess the performance of the alternatives with respect to each criterion directly. The alternatives are evaluated independently of each other.

From a methodological point of view, the scoring systems discussed in Section 6 are equivalent to the points scoring systems used in sports contests such as Formula One or biathlon. Several questions can be interesting in both areas:

– What happens with the ranking if a competitor is disqualified? In sports, it is usually due to doping, but the IOC has also disqualified two winners because of noncompliance with the rules.
– How are the set of competitions and their importance chosen? For instance, the EVOO World Ranking does not announce what criteria a competition should satisfy or take into account. Analogously, the assignment of the scores remains obscure except for the unique maximum given to IOC Mario Solinas.
– Does the scoring system satisfy reasonable theoretical requirements? Is it monotonic, that is, a better performance could not decrease the final score? How likely is it that a Condorcet winner does not win? What is the probability that the number one in the world ranking has not won any competition?

According to our knowledge, these questions have not been studied in the food science literature yet. However, they are widely discussed in sports. Let us mention two recent studies. Kondratev et al. (2023) derive a one‐parameter family of scoring rules that satisfies two independence principles: the aggregated ranking should remain unchanged if a unanimously strong or weak competitor is removed. The recent decision of IOC (IOC 2023a) shows the relevance of this problem in EVOO competitions. Csató (2023) investigates the trade‐off in Formula One between two risks: (1) when the world champion becomes known before the last contests; (2) when the world champion does not win any race. Both dangers are present in food competitions since a producer may refuse to enter a contest if it has already obtained the EVOO of the year title, and it is also not preferred if the top position in a world ranking can be secured without winning a first prize in any food competition.

Currently, the results of individual competitions are usually aggregated by points scoring systems as discussed in Section 6. However, this is not the only possibility if the products are evaluated in more than one competition. Then the results of a competition can be regarded as pairwise comparisons of the products, which can be aggregated into a pairwise comparison matrix based on several competitions. The competitions may get different weights according to their prestige, and the pairwise comparisons derived from their results might depend on the size of differences (e.g., the winner of the first prize is judged two times better than the winner of the second prize but three times better than the winner of the third prize). This technique has been applied to rank top players in sports who have never played against each other (Bozóki et al. 2016; Chao et al. 2018; Temesi et al. 2024), as well as to obtain university rankings based on the revealed preferences of the students (Csató and Tóth 2020). Theoretical findings may also provide information about which competition should be entered by a given product in order to maximize the reliability of the final ranking (Szádoczki et al. 2022).

Scores or rankings can also be calculated from ordinal or cardinal paired comparisons as discussed in the next section.

The advantage of classification is that it is simple, fast, easy to understand, and well suited for discrete decisions (e.g., quality control). Statistical inferences can be drawn about differences between products based on the frequency of categories. On the other hand, classification does not provide information on the differences between classes, on their extent, and on the strength of preferences. Grading is easy to understand, using medians to allow group comparisons of products and to determine quality levels. However, the method is not suitable for detecting small differences and, in the case of a large number of identical ranks, special statistical procedures are needed. Ranking is simple to implement, does not require an absolute assessment, and is suitable for detecting small differences between samples. However, ranking only shows relative differences; it does not reflect the magnitude of preferences. Identical values are not allowed, and ranking many samples can lead to sensory fatigue. Categorizing is an effective method of ranking into homogeneous groups, where nonparametric methods are well suited to compare multiple samples. The disadvantage of the method is that some categories may overlap, and their definition may be subjective, and it does not provide information on the extent of differences. The advantage of scoring is that its quantitative results are well suited for statistical analysis. It has the advantage of being applicable to all types of assessors, has a high degree of accuracy, can be combined with other methods, and can be used to determine not only the ranking but also the extent of differences between individual attributes. However, the use of the scale may vary from one assessor to another, and the results of scoring may be sensitive to changes in the set of products compared.

## 8 Rankings Based on Pairwise Comparisons

Formal models of *ordinal* preferences appeared in the work of Ramon Llull (Llull 2001; Hägele and Pukelsheim 2001) focusing on voting systems in the 13th century. Half a millennium later, Marquis de Condorcet proposed the same concept of *majority preference* between two candidates, that is, when more voters prefer one to another than vice versa. Condorcet identified that majority preferences can be cyclical/nontransitive (Black 1958). Arrow's impossibility theorem clarified the limits of voting models based on ordinal preferences: if the number of alternatives is at least three, then there exists a preference profile for any method of preference aggregation such that the group decision fails to satisfy some natural requirements formalized as axioms. Naturally, Arrow's theorem does not imply that every method must fail for every preference profile, but the search for a universal ideal voting method is proved to be hopeless. Furthermore, since the usual approach is setting the voting method first and then collecting individual votes, it cannot be guaranteed that the current preference profile would not fall into the ones “hacking” the desired properties. The only exception is the case of two alternatives (e.g., yes/no), where the simple majority rule is the only acceptable (again, satisfying a set of reasonable axioms) method (May 1952).

*Cardinal* preferences are given by numerical intensities. A sufficiently large difference of scores given by a decision maker can compensate for several smaller differences, in the opposite way, given by others, and vice versa. It can also be a source of possible manipulation, as it happened in several sport competitions. Before the truncated mean (the highest and lowest scores are not considered) was introduced, a jury member could easily overrate a favored athlete to make them win.

**FIGURE 5:**
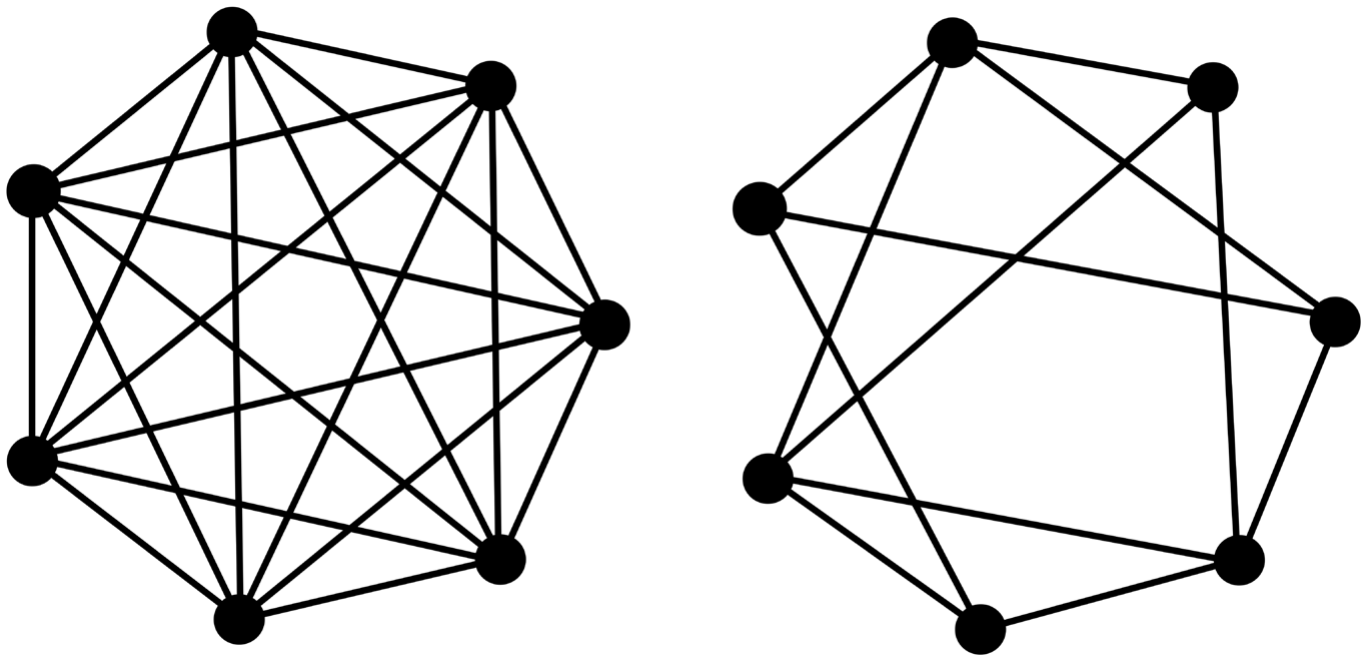
Complete (left) and incomplete (right) sets of pairwise comparison of seven items.

Pairwise comparisons on a ratio scale (how many times an alternative is better or a criterion is more important than the other one) are used in for example, the popular Analytic Hierarchy Process (AHP) methodology. The decision makers provide cardinal information, and cardinal weight vectors are calculated. Decision support systems such as Expert Choice, SuperDecisions, webHIPRE, and PriEst also contributed to that AHP is widely used in practice. Models based on incomplete pairwise comparisons, when not the entire set of all possible pairs is compared, offer a still wider range of applications, for example, ranking thousands or millions of alternatives. The structure of known paired comparisons can be represented by a graph (Figure 5), as in, for example, Bozóki et al. (2016). It also offers a wide range of interpretations (Csató 2015).

**FIGURE 6:**
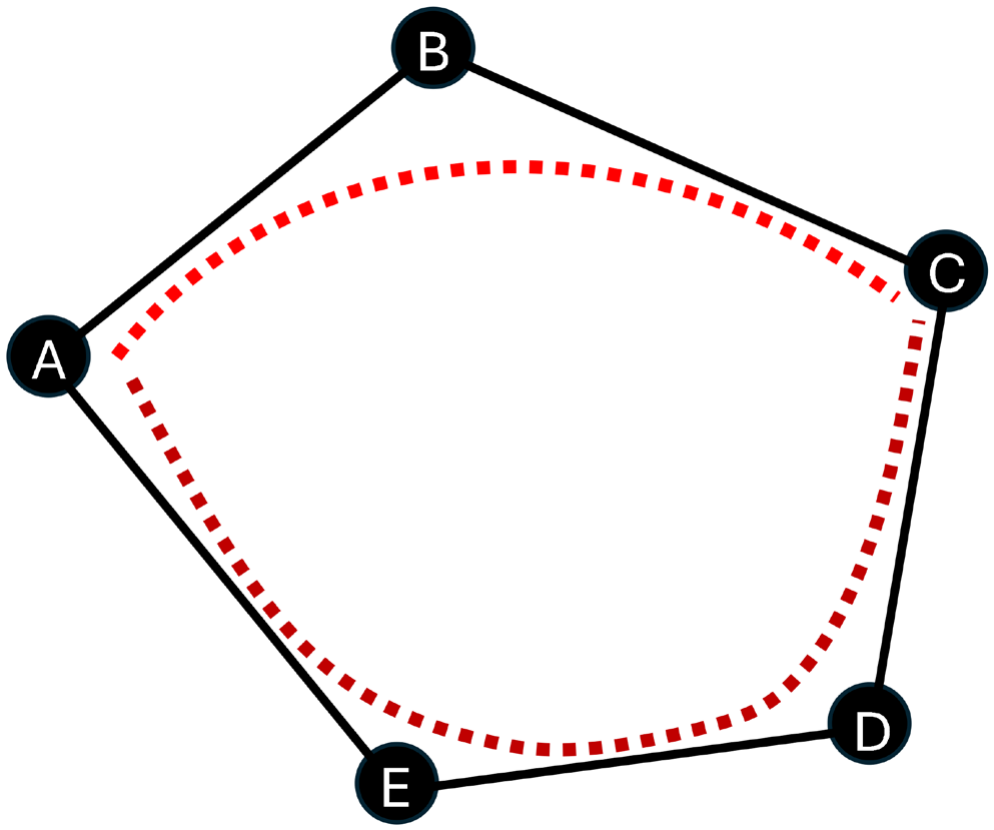
Direct (black) comparison of pairs AB, BC, CD, DE, EA; and indirect (red and dark red dotted) comparisons of A and C.

Quantification of preferences based on a graph theoretical approach builds on not only the comparisons themselves but their structure as well. Indirect comparisons (via chains of direct ones) may yield better approximations (Figure 5). Although we cannot assume cardinal or even ordinal transitivity in general, it is usually still better to have some indirect information than no information at all. PageRank uses the graph of links (ordinal information) to calculate the weight of importance/relevance of vertices—originally websites, but the idea applies to any kind of interlinked objects to be ranked (scholars, scientific journals, universities, assessors' performance, etc.). Interestingly, but at the same time reasonably, the most important/relevant websites are not necessarily the most frequently linked ones. They can be among the most important ones if they are linked by fewer, but also very important sites (Figure 6).

From the early 2000s, the practical implementation of the network's empirical research started to grow, and the Hungarian‐American physicist László Barabási played a prominent role in its implementation, international recognition, and dissemination. Barabási and Frangos (2014) analyzed networks related to several kinds of human activities and relations and found that their structural properties are very similar. Scale independence and a low number of vertices with extremely high degrees (representing importance or influence) are almost always present, unlike in random networks.

Based on international and regional culinaries of the world, weighted bipartite networks of flavors are identified, with statistically significant edges/links retained in visualization (network's extracted backbone). The network consists of two kinds of nodes: ingredients in the recipes and ingredient flavor compounds. The edges/links represent the number of flavor compounds shared among the ingredients (Ahn et al. 2011; Makinei and Hazarika 2022). Databases on food smells, tastes, flavors, and chemical components, recipe collections, etc., have laid the foundation for a data‐driven approach to food. Computational gastronomy is a part of data science that combines food, data, and the power of computation to achieve data‐driven food innovations (molecular basis of flavors and aromas, flavor pairing, food pairing, new recipe generation, food for special diets, culinary patterning) (Ahnert 2013; Goel and Bagler 2022).

Sensory and instrumental analytical measurements of alcoholic beverages can be connected: aroma networks link the chemical and sensory attributes of white and red wines and identify the volatile chemical compounds that form the sensory characteristics that determine aromas. As a result of the analysis, the fingerprints of the wines can be determined, and the similarity of the wines can be characterized by the thickness (weight) of the edges of the graph, that is, the frequencies of the volatile compounds in the wines. This aroma network approach can be used in food quality control by including additional foods (Petronilho et al. 2020).

Nestrud et al. (2011, 2012) have demonstrated the principle of supercombinatoriality for both individuals (salad ingredient combinations) and groups (pizza toppings), that food combinations that are fully compatible per pair of more than two items are more compatible than combinations that are not fully compatible per pair. The collection and aggregation of individual preferences on a subset of items can lead to remarkable predictions. Nestrud et al. (2011) look for maximal cliques (complete subgraphs), as well as noncliques (empty subgraphs) in a graph, where the 25 vertices represent popular salad ingredients and an edge between two vertices indicates the compatibility of the given pair. For example, the combination of Bell pepper, Black olive, Blue cheese, Broccoli, Chicken, Cucumber is found attractive, while the combination of Avocado, Bacon, Bell pepper, Broccoli, Corn, Cucumber is not worth preparing. Nestrud et al. (2012) used a prescreening questionnaire to identify the 25 most frequently chosen pizza toppings (if price were not a factor). Then they rated the preferences of consumers for pizza toppings, based on which they predicted the pizzas (1–6 toppings) that would be most preferred by the whole group. The study highlights that potentially preferred food combinations can be identified by using consumer responses and a graph‐theoretic approach.

Outranking methods such as PROMETHEE (Brans et al. 1986) are also based on pairwise comparisons. Preference functions map the difference between the performance of two alternatives with respect to a criterion to a preference value in the interval [0, 1]. The decision maker is responsible for the choice of preference functions and their (cardinal) parameters. Criterion‐wise preference values are aggregated to cardinal total scores (net flow values), implying a ranking as well. PromCalc, Decision Lab 2000, Visual Promethee are among the most popular software of this methodology.

The importance of a criterion, as it has no absolute scale, is naturally derived or estimated through comparisons, ordinal (e.g., Guilford, SMARTER), cardinal (trade‐off), or both (e.g., Churchmann‐Ackoff, SMART). Platforms such as XLSTAT, Decision Deck, Decisionarium, and the collection of the International Society of Multiple Criteria Decisions Making (MCDM) (https://www.mcdmsociety.org/content/software‐related‐mcdm‐0) offer multi‐criteria decision software and support the choice among them, which is itself a multi‐criteria decision problem. The process of revealing as well as of displaying cardinal preferences is often supported by visual tools such as single or multilevel pie charts (Expert Choice, XLSTAT), sliders (CompuSense, Fizz, RedJade), dynamic bar charts (Expert Choice), and radar/spider charts (XLSTAT).

The above mentioned methods and implementations uncover that, in the case of a particularly high number of items to be ranked, the aggregation of individual choices can reflect the global preferences relatively well even if each evaluator provides information on a negligibly small subset.

The main advantage of ranking based on pairwise comparisons is that working with incomplete data can substantially reduce the burden of the assessors since it is not necessary to compare all possible pairs of samples. A wide range of supporting software tools is available for these methods, which are usually able to handle both ordinal (sequence) and cardinal (intensity) information. However, the preferences may be inconsistent, which can cause difficulties in their interpretation. In cardinal methods, a single decision maker might have a disproportionate influence on the outcome (e.g., manipulation of scores in sports competitions). Some methods, such as AHP or PROMETHEE, can be complex to learn and use, especially for nonexperts.

## 9 Future Challenges and Trends

Based on the literature reviewed, the following future trends can be expected:

1. Different methods and software from other disciplines—decision theory, economics, mathematics, operations research—will be adapted and disseminated in order to better understand consumer preferences and to explore complex consumer choices. The use of pairwise ranking, multi‐criteria decision modeling, graph theory, etc. will become more widespread in food science, which will integrate recent developments of preference modeling, utility decomposition, and identification (principal component analysis, conjoint analysis).
2. The proliferation and practical applications of graph theoretical methodologies have been triggered by research issues related to networks and their interconnection systems but were in turn enabled by the conditions created by large data sets and structured databases. Their spread has been greatly facilitated by visual representation, visualization of links, network points, and relationships. In sensory sciences, graph theory plays currently only a marginal role, but the related approaches of visual representation can bring a novel insight into previously commonly used methodologies (BIB design), and its role is expected to increase in the developments with large data sets (computational gastronomy, sensometry, product development, analysis of consumption patterns, cross‐cultural studies, effects of influencer content on food choice).
3. Sensory research is supported by standard methods, and among the methods of experimental design, methodologies for comparative purposes are integrated, such as the Latin Square design (ISO 11132 2021) and the Balanced Incomplete Block design (ISO 29842 2011, 2015). At the same time, methods of experimental design for screening and response surface purposes are used in practice in many places and are expected to be integrated into standards. Systematic application of these experiment design methodologies will allow more efficient and less costly (time, energy, money) studies to be carried out that still meet the objectives of the experiment.
4. Sensory product competitions are currently biased by a number of sensory defects and are strongly tradition‐bound and product‐specific, which determine the range of products tested, the testing protocol, the evaluation procedure, and the prizes awarded. At the same time, what started as relatively obscure local competitions have increasingly become large, regional, and then international events, marketing and communication tools worldwide. In addition to the heterogeneous nature of competitions, there is a need for the comparability of competitions and their results, both from the consumer and from the commercial and expert side. Initiatives have been taken to solve this problem (see Sense‐Award in Section 6), but at the same time, the comparison of current competition results is a major challenge for researchers (data availability, heterogeneous evaluation protocols, different levels of judges' qualifications, filtering), and the proposed solutions suggest a new research direction.
5. Even though the rules of international food competitions show a high degree of heterogeneity (properties, weights, aggregation methods) due to their product specificity and traditions, these rules are unlikely to be standardized in the near future. Nevertheless, since the rules of international food competition are usually not sufficiently specified, contain a mixture of objective and subjective elements, and suffer from internal contradictions in their scoring systems, the study of these potential weaknesses poses an important research direction.
6. The reliability of the results from international scoring sensory competitions is based on the implicit assumption that the decisions of the assessors are internally consistent. However, this crucial condition is rarely tested and fed back into the evaluating system. The necessary methods and good practices should be developed in the near future.
7. Prizes allocated in international food competitions can generate greater market penetration and become a profit motive beyond the prestige aspect, even though the knowledge of consumers about the prizes is expected to remain minimal.
8. The reliability of assessors' evaluations can be increased by using simpler (e.g., ordinal) methods rather than scoring. Minimizing the number of sensory steps or comparisons can also be beneficial with respect to both mental and sensory fatigue. Thus, the use of ordinal scales, in particular, paired comparisons, will gain prominence at the expense of complex scoring systems.
9. In almost all cases, the tests completed by the consumers contain incomplete answers that are not adequately managed. The findings of preference estimation of incomplete matrices generated by pairwise comparisons could potentially be integrated into consumer test applications.
10. Since computer‐based technologies are becoming widespread for the collection and aggregation of individual preferences, more intelligent methods can be developed for preference elicitation and evaluation for both sensory tests and competitions. An example is adaptive questioning, where the refinements of comparative and ranking questions are based on the answers to the previous questions.
11. Recommendation systems based on food consumption and individual choices, using deep learning algorithms trained on big data sets, will become increasingly important in shaping consumer preferences. Mobile applications will play a key role in building databases.
12. Recent trends in the elicitation of individual preferences: customization, personalization, bottom‐up initiatives, crowd‐based evaluation, and crowd‐funding are alternatives to traditional product development, inherently including that of foods and drinks.
13. With the development of sensors and the integration of physiological responses to different stimuli (eye movements, pupil dilation, brain waves, skin resistance, heart rate variability), emotional analytics (facial muscles, facial expressions), the latent, unconscious factors of consumer perception and decision‐making can be determined. Consumer search patterns can also be revealed, and the accuracy of response prediction will improve.
14. The integration of psycho‐physical responses could significantly change the way food is sold in the retail and the Hotel, Restaurant, Catering (HORECA) sector (experimentally focused food consumption/shopping, smart stores, robotics, redesigned store routes, modified shelf displays, touch‐free solutions, virtual restaurant environments, optimized menus, innovative food pairings, etc.).

At present, the opinions/preferences of a few hundred people determine the choice of products for millions of people in the general practice of food development. In the future, however, this will likely be reversed: the dynamically growing and ever‐expanding, increasingly specified large consumer databases will strongly influence individual choices. At the same time, the demand for food products tailored to individual preferences, age, gender, and health will increase. In standard sensory testing, product attributes are ranked by individual attributes, while in the practice of multidimensional optimization of product development. Currently, the competition is usually between different brands of products in food markets, but this is gradually becoming a competition between distribution channels, databases, information, algorithms, and recommendation systems.

## 10 Conclusions

The international standardization (ISO) system has been established over the recent decades. However, the process of integrating opinions from experts of national member bodies in several stages, based on their feedback, is long. No acceleration of the standardization process is forecast. The integration of methodologies in response to market needs can only be achieved after a significant time lag, usually years. The standard (ISO) methods for classical ranking tests have been developed, and new statistical methods in addition to the standard tests, new visualization possibilities are being introduced in statistical software. The development and integration of BIBDs for a larger number of items and for a larger number of reviewers into the international standard (ISO 29842 2011, 2015) is expected.

The field of mathematical statistics has already contributed to consumer and sensory sciences with a number of methods, including standard ranking tests (nonparametric statistical methods of evaluation, for example, Spearman correlation coefficient, Sign test, Wilcoxon test, Friedman test, Page test and post hoc tests, BIBD experimental designs and statistical evaluation). This is further evidenced by the fact that the international ranking (ISO 8587 2006) and the BIBD (ISO 29842 2011, 2015) standard use and refer to many statistical sources. However, new challenges in the food industry and in food research require the integration of the latest methodologies. The first step towards its widespread dissemination is to learn, test, adapt, and accept new or little known methodologies:

Preference modeling and quantification are key issues in decision and sensory sciences. Both areas require true, unbiased individual responses as well as their appropriate aggregation to group decisions. However, the time and attention of decision makers, experts, or assessors remain a bottleneck: efficiency does not allow for asking unnecessary questions without substantial information, or the involvement of more respondents than necessary. Automated sensory also reduces the assessors' efforts and can increase the reliability of the data. Nonetheless, the human factor cannot be eliminated, since the products are developed for them, according to their needs and taste. Customization is supported by data, collected by direct and indirect methods.

Decision and voting theory includes several axiomatic results: a set of desired properties is considered, depending on the particular characteristics of the problem. Methods satisfying all the axioms can be recommended because the possibility of an unexpected failure, often occurring as a counterintuitive outcome, is excluded.

Competitions provide immediate additional information. In order to shorten the decision process, consumers apply heuristics like the assumed additional utility induced by an award. The ranking itself can be regarded as a common heuristic. Competitions in sport or food are similar from a methodological point of view: almost all of them are multi‐criteria problems, evaluated by one or, more typically, more decision makers. Thus, the recent results and trends in sports competitions, especially tournament design, can be interesting for food science as well.

## Author Contributions

**László Sipos:** conceptualization (equal), funding acquisition (equal), investigation (equal), methodology (equal), software (equal), supervision (equal), validation (equal), visualization (equal), writing – original draft (equal), writing – review and editing (equal). **Zsófia Galambosi:** formal analysis (equal), methodology (equal), software (equal), visualization (equal), writing – original draft (equal), writing – review and editing (equal). **Péter Biró:** funding acquisition (equal), methodology (equal), validation (equal), visualization (equal), writing – original draft (equal), writing – review and editing (equal). **László Csató:** conceptualization (equal), funding acquisition (equal), methodology (equal), supervision (equal), validation (equal), visualization (equal), writing – original draft (equal), writing – review and editing (equal). **Sándor Bozóki:** conceptualization (equal), methodology (equal), software (equal), supervision (equal), validation (equal), visualization (equal), writing – original draft (equal), writing – review and editing (equal).

## Ethics Statement

The authors have nothing to report.

## Supporting information


**Data S1:** fsn370684‐sup‐0001‐supinfo.docx.

## Data Availability

All data and processed results are presented in the manuscript and the Supporting Information.
