# Supplementary material for: Trends and Directions of Preference Elicitation and Assessment in Food Science: Single‐, Pair‐, and Multi‐Criteria Ranking Methods
Source: Food Sci Nutr. 2025 Jul 31;13(8):e70684. doi: 10.1002/fsn3.70684 (PMC12311391; doi:10.1002/fsn3.70684)
Supplement: Supplementary file 1 — Data S1: fsn370684‐sup‐0001‐supinfo.docx. [file FSN3-13-e70684-s001.docx]

**Table 1.** Wine competitions around the world (continent, website, organising country)

| **American competitions** | **Website** | **Organising country** |
| --- | --- | --- |
| California State Fair Commercial Wine Competition | <https://calexpostatefair.com> | USA |
| California State Fair Home Wine Competition | <https://calexpostatefair.com> | USA |
| Colorado State Fair Commercial Wine Competition | <https://coloradostatefair.com> | USA |
| Colorado State Fair Hobby Wine Competition | <https://coloradostatefair.com> | USA |
| Dan Berger’s International Wine Competition | [https://www.dbiwc.com](https://www.dbiwc.com/) | USA |
| El Dorado County Fair Open Wine Competition | <https://eldoradocountyfair.org> | USA |
| El Dorado County Fair Homemade Wine Competition | <https://eldoradocountyfair.org> | USA |
| Experience Rosé Wine Competition | <https://www.winecompetitions.com> | USA |
| Harvest Challenge | <https://www.winecompetitions.com> | USA |
| International East Meets West Wine Challenge | <https://www.winecompetitions.com> | USA |
| International Eastern Wine Competition | <https://www.winecompetitions.com> | USA |
| International Women’s Wine Competition | <https://www.winecompetitions.com> | USA |
| Los Angeles International Wine Competition | <https://fairplex.com> | USA |
| Los Angeles Invitational Wine & Spirits Challenges | <https://lainvitational.com> | USA |
| New York World Wine & Spirits Competition | <https://thetastingalliance.com> | USA |
| Orange County Fair Commercial Wine Competition | [https://ocws.org](https://ocws.org/) | USA |
| Orange County Fair Home Wine Competition | [https://ocws.org](https://ocws.org/) | USA |
| Pacific Rim International Wine Competition | [https://pacificrimwinecomp.com](https://pacificrimwinecomp.com/) | USA |
| San Diego International Wine & Spirits Challenge | [https://www.sandiegowinechallenge.com](https://www.sandiegowinechallenge.com/) | USA |
| San Francisco Chronicle Wine Competition | <https://winejudging.com> | USA |
| San Francisco International Wine Competition (SFIWC) | <https://thetastingalliance.com> | USA |
| Sommeliers Choice Awards | [https://sommelierschoiceawards.com](https://sommelierschoiceawards.com/) | USA |
| Sunset International Wine Competition | <https://www.winecompetitions.com> | USA |
| The Press Democrat North Coast Wine Challenge | <https://www.pdncwc.com> | USA |
| USA Wine Ratings | [https://usawineratings.com](https://usawineratings.com/) | USA |
| West Coast Wine Competition | <https://www.winecompetitions.com> | USA |
| Wine & Spirits Wholesalers of America | [https://www.wswa.org](https://www.wswa.org/) | USA |
|  |  |  |
| **European competitions** | **Website** | **Organising country** |
| 50 Great Canned Wines of the World | <https://www.winepleasures.com> | Spain |
| 50 Great Cavas | [https://www.winepleasures.com](https://www.winepleasures.com/contest/50-great-cavas-2022-awards-scores/) | Spain |
| 50 Great Red Wines of the World | <https://www.winepleasures.com> | Spain |
| 50 Great Sparkling Wines of the World Competition | <https://www.winepleasures.com> | Spain |
| 50 Top Rosé Wines of the World | <https://www.winepleasures.com> | Spain |
| Alliance du Monde | <https://www.alliances-du-monde.com> | France |
| Balkans International Wine Competition | <http://www.balkanswine.eu/en/> | Romania |
| Berliner Wine Trophy | [https://www.wine-trophy.com](https://www.wine-trophy.com/) | Germany |
| Brazil Wine Challenge | <https://brazilwinechallenge.com.br> | Brazil |
| Challenge des Marques (Le Challenge des Vins 100% MDD) | [https://www.challengedesmarques.com](https://www.challengedesmarques.com/) | France |
| Challenge des Terroirs | [https://challenge-des-terroirs.com](https://challenge-des-terroirs.com/) | France |
| Challenge International du Vin | <https://www.challengeduvin.com/en/> | France |
| Challenge Millésime Bio | <https://www.challenge-millesime-bio.com/en> | France |
| Champagne & Sparkling Wine World Championships (CSWWC) | [https://www.champagnesparklingwwc.co.uk](https://www.champagnesparklingwwc.co.uk/) | UK |
| Chardonnay du Monde | [https://www.chardonnay-du-monde.com](https://www.chardonnay-du-monde.com/) | France |
| Citadelles du Vin | <https://www.citadellesduvin.com> | France |
| Comité Interprofessionnel des Vins du Jura | <https://www.jura-vins.com> | France |
| Concours International Des Cabernets (CIDC) | <https://www.concourscabernets.com/en> | France |
| Concours 100 % Carignan | <https://www.igp-herault.fr> | France |
| Concours Anivin de France Best Value Selection | <https://www.vindefrance.com/wines/pro> | France |
| Concours AOC Côtes du Rhône Village et Crus | <https://vinsobres.fr> | France |
| Concours Général des Vins des Courtiers Assermentés de France | <https://courtiers-assermentes.org> | France |
| Concours d’Aix-en-Provence | <https://chateauberne-vin.com> | France |
| Concours de Bordeaux-Vins d’Aquitaine | [https://www.concours-de-bordeaux.com](https://www.concours-de-bordeaux.com/) | France |
| Concours Départemental des Vins – Clermont Ferrand | <https://www.cotesdauvergne.com> | France |
| Concours Départemental des Vins de l’Isère | <https://www.lapogeeduvin.com> | France |
| Concours Départemental des Vins | <https://centre-valdeloire.chambres-agriculture.fr> | France |
| Concours des Burgondia | [https://www.burgondia.com](https://www.burgondia.com/) | France |
| Concours des Chardonnay et Pinot Noir | <https://concours-chardonnay-pinot.com/en> | France |
| Concours des Crus du Beaujolais – Coupe Victor Pulliat | <https://chiroubles-wine.com> | France |
| Concours des Grands Vins de France à Mâcon | [https://www.concours-salons-vins-macon.com](https://www.concours-salons-vins-macon.com/) | France |
| Concours des Grands Vins du Beaujolais | [https://www.concoursbeaujolais.com](https://www.concoursbeaujolais.com/) | France |
| Concours des Ligers | <https://www.concoursdesligers.fr/le-palmares> | France |
| Concours Saveurs Nouvelle-Aquitaine | [https://www.saveurs-nouvelle-aquitaine.fr](https://www.saveurs-nouvelle-aquitaine.fr/) | France |
| Concours des Vins AOC Ventoux | <https://aoc-ventoux.com> | France |
| Concours des Vins à Orange | [https://www.concoursdesvins.fr](https://www.concoursdesvins.fr/) | France |
| Concours des Vins - Avignon | [https://www.concoursdesvins-avignon.com](https://www.concoursdesvins-avignon.com/) | France |
| Concours des Vins de Gaillac et des Vins du Tarn | [https://www.vins-gaillac.com](https://www.vins-gaillac.com/?legal=1) | France |
| Concours des Vins de la Coopération Occitanie | [https://concoursdelacooperation.fr](https://concoursdelacooperation.fr/) | France |
| Concours des Vins de la Foire de Brignoles | <https://foiredebrignoles.fr> | France |
| Concours des Vins de la Région de Bergerac et Duras | <https://www.fv-bergerac.fr> | France |
| Concours des Vins de la vallée de l’Hérault | [https://www.vins-vallee-herault.fr](https://www.vins-vallee-herault.fr/) | France |
| Concours des Vins de Nîmes | <http://jeunesagriculteursdugard.fr> | France |
| Concours des Vins de Provence | [https://www.vinsdeprovence.com](https://www.vinsdeprovence.com/) | France |
| Concours des Vins des Coteaux du Quercy | <https://vins-coteaux-quercy.fr> | France |
| Concours des Vins des Jeunes Agriculteurs du Var | <https://jeunesagriculteurs83.fr> | France |
| Concours des Vins de Tulette | [https://concoursdesvinstulette.com](https://concoursdesvinstulette.com/) | France |
| Concours des Vins d’Orange | <https://www.sylla.fr> | France |
| Concours des vins du Sud-ouest – France | <https://www.concours-vins-sudouest-france.fr> | France |
| Concours des Vins du Val de Loire | <https://www.vinsvaldeloire.fr/fr> | France |
| Concours des vins Elle à table | [https://trophees-vins.elle.fr](https://trophees-vins.elle.fr/) | France |
| Concours des vins IGP de France | <https://www.concoursnationaligp.vin> | France |
| Concours des vins IGP du Lot | <https://cotesdulot.fr> | France |
| Concours des Vins IGP Val de Loire | <https://www.concoursvinsvaldeloire.fr> | France |
| Concours des Vins Jury Consommateurs | <https://www.aocvacqueyras.com> | France |
| Concours des Vins: le Palmarés | <https://www.vigneron-independant.com> | France |
| Concours des vins Mâconnais Beaujolais | [http://concours-des-vins-maconnais-beaujolais.e-monsite.com](http://concours-des-vins-maconnais-beaujolais.e-monsite.com/) | France |
| Concours des Vins Saumur | [https://www.vins-de-saumur.com](https://www.vins-de-saumur.com/) | France |
| Concours des Vins Terre de Vins | [https://concours.terredevins.com](https://concours.terredevins.com/) | France |
| Concours Général Agricole | [https://www.concours-general-agricole.fr](https://www.concours-general-agricole.fr/) | France |
| Concours Général des Vins des Courtiers Assermentés de France | <https://courtiers-assermentes.org> | France |
| Concours International Best Wine in Box | <https://best-wine-in-box.com/fr> | France |
| Concours International de Lyon Wine Competition | <https://www.concourslyon.com/en> | France |
| Concours International des Produits Biologiques | <https://concoursbio.com/en> | France |
| Concours Interprofessionnel des Vins de Corbières | <https://vins-corbieres.com/fr> | France |
| Concours Mondial de Bruxelles | <https://concoursmondial.com/en/> | Belgium |
| Concours Mondial des Feminalise | [https://www.feminalise.com](https://www.feminalise.com/) | France |
| Concours Mondial du Savagnin | [http://www.concoursmondialdusavagnin.com](http://www.concoursmondialdusavagnin.com/) | France |
| Concourss Mondial du Sauvignon | <https://sauvignonselection.com/fr/> | France |
| Concours National de Paris | [https://a-vgf.com](https://a-vgf.com/) | France |
| Concours National des Crémants | [https://cremants.com](https://cremants.com/) | France |
| Concours National des Vins IGP de France | [https://www.vinigp.fr](https://www.vinigp.fr/) | France |
| Concours Touraine Primeur | <https://www.vintouraine.com> | France |
| Cyprus Wine Competition | <https://www.oiv.int/index.php/> | Cyprus |
| Decanter World Wine Awards (DWWA) | <https://www.decanter.com/enter/> | UK |
| Distinction Saint Vincent des Vins Mâcon | [https://www.vins-macon.com](https://www.vins-macon.com/) | France |
| Effervescents du Monde | [https://www.effervescents-du-monde.com](https://www.effervescents-du-monde.com/) | France |
| Expo-Vall’ | <https://www.expovall.fr> | France |
| Expression des Vignerons Bio de Nouvelle Aquitaine | [http://www.vigneronsbionouvelleaquitaine.fr](http://www.vigneronsbionouvelleaquitaine.fr/) | France |
| FIWA - France International Wine Awards | [https://www.fiwa.fr](https://www.fiwa.fr/) | France |
| Grenaches du Monde | <https://www.grenachesdumonde.com/en/> | France |
| Glass of Bubbly Awards | <https://glassofbubbly.com> | UK |
| Golden League | <https://www.wine-trophy.com> | Germany |
| Grand Concours des Vins d’Alsace de Colmar | <https://www.vinsalsace.com/fr> | France |
| Grand Prix Clémence Lefeuvre | [https://prixclemence.fr](https://prixclemence.fr/) | France |
| International du Gamay | <https://www.concoursgamay.com/en> | France |
| International Wine Challenge (IWC) | [https://www.internationalwinechallenge.com](https://www.internationalwinechallenge.com/) | UK |
| La Percée du Vin Jaune | <https://www.percee-du-vin-jaune.com> | France |
| Le Concours des Lauréades | <http://www.courtiersenvinsdeparis.org> | France |
| Le Mondial des Vins Blancs Strasbourg | [https://www.mondial-vins-blancs.com](https://www.mondial-vins-blancs.com/) | France |
| Le Tastevinage | [https://tastevinage.fr](https://tastevinage.fr/) | France |
| Les rosés au féminin | <https://cotesdulot.fr> | France |
| London Wine Competition | <https://londonwinecompetition.com/en/> | UK |
| Muscats du Monde | [https://www.muscats-du-monde.com](https://www.muscats-du-monde.com/) | France |
| Palmarés des vins Sylla Médailles | <https://www.sylla.fr> | France |
| Portugal Wine Trophy | <https://www.wine-trophy.com> | Portugal |
| Prix d’Excellence | <https://www.concours-general-agricole.fr> | France |
| Prix d’Excellence des Vins des Hautes-Côtes | <https://www.lesbuvologues.com> | France |
| Prix Plaisir Bettane & Desseauve | <https://prixplaisir.bettanedesseauve.com> | France |
| Salon des Vins et terroirs de thouars | <http://salon-vins-terroirs-thouars.org/index.php/fr/> | France |
| Sigille des Vins d’Alsace | <https://confrerie-st-etienne.alsace> | France |
| Soleils des vins de pays du Var | <https://www.syndicatdesvigneronsduvar.com/index.php> | France |
| Syrah du Monde | [https://www.syrah-du-monde.com](https://www.syrah-du-monde.com/) | France |
| The DB & SB Spring Blind Tasting | <https://www.globalwinemasters.com> | UK |
| The Global Cabernet Sauvignon Masters | <https://www.globalwinemasters.com> | UK |
| The Global Carmenère Masters | <https://www.globalwinemasters.com> | UK |
| The Global Chardonnay Masters | <https://www.globalwinemasters.com> | UK |
| The Global Low & No Alcohol Wine Masters | <https://www.globalwinemasters.com> | UK |
| The Global Malbec Masters | <https://www.globalwinemasters.com> | UK |
| The Global Organic & Vegan Wine Masters | <https://www.globalwinemasters.com> | UK |
| The Global Pinot Grigio/ Pinot Gris Masters | <https://www.globalwinemasters.com> | UK |
| The Global Pinot Noir Masters | <https://www.globalwinemasters.com> | UK |
| The Global Riesling Masters | <https://www.globalwinemasters.com> | UK |
| The Global Rosé Masters | <https://www.globalwinemasters.com> | UK |
| The Global Sauvignon Blanc Masters | <https://www.globalwinemasters.com> | UK |
| The Global Syrah Masters | <https://www.globalwinemasters.com> | UK |
| The Grenache Masters | <https://www.globalwinemasters.com> | UK |
| The Prosecco Masters | <https://www.globalwinemasters.com> | UK |
| The Rioja Masters | <https://www.globalwinemasters.com> | UK |
| Thessaloniki Wine & Spirits Competition | <http://www.tiwc.gr/index.php?lang=en> | Greece |
| Trophées des Vins de Savoie | <https://www.adrien-vacher.fr> | France |
| Trophée du Savoir-faire Vigneron Vauclusien | <https://www.sylla.fr/fr/> | France |
| Trophée Beaujolais Nouveau | [http://www.trophee-beaujolais.com](http://www.trophee-beaujolais.com/) | France |
| Vinalies France | <https://www.vinalies-nationales.fr/en/> | France |
| Vinalies Internationales | <https://www.vinalies-internationales.com/en/> | France |
| Vinalies Mondial du Rosé | <https://www.mondial-du-rose.com/en/> | France |
| Vinalies Occitanie | <https://cgvlr.oenologuesdefrance.fr/fr/> | France |
| VINARIUM International Wine Contest | <http://www.iwcb.ro/en/> | Romania |
| Vinitaly Awards | <https://www.vinitaly.com/en/> | Italy |
| Vins de Lorrains | <https://vins-de-lorraine.fr> | France |
|  |  |  |
| **Asian competitions** | **Website** | **Organising country** |
| Asia International Wine Competition | [https://asiainternationalwinecompetition.com](https://asiainternationalwinecompetition.com/) | Singapore |
| Asia Wine Trophy | <https://www.wine-trophy.com> | South-Korea |
| Cabernet Sauvignon Asia Masters | <https://www.globalwinemasters.com> | Hong Kong |
| The Asian Pinot Noir Masters | <https://www.globalwinemasters.com> | Hong Kong |
| The Asian Syrah Masters | <https://www.globalwinemasters.com> | Hong Kong |
| The DB Asia Winter Tasting | <https://www.globalwinemasters.com> | Hong Kong |
|  |  |  |
| **Oceanic competitions** | **Website** | **Organising country** |
| Melbourne Royal Wine Awards | <https://www.melbourneroyal.com.au> | Australia |
| Perth Royal Wine Awards | <https://perthroyalfoodawards.raswa.org.au> | Australia |
| Royal Queensland Wine Awards | [https://www.rqa.com.au](https://www.rqa.com.au/) | Australia |
| Sydney International Wine Competition | [https://sydneywinecomp.com](https://sydneywinecomp.com/) | Australia |
|  |  |  |
| **International competitions** | **Website** | **Organising country** |
| International Wines & Spirits Competition | [https://www.iwsc.net](https://www.iwsc.net/) | more countries in a year |

**Table 2.** Beer competitions around the world (continent, website, organising country)

| **American competitions** | **Website** | **Organising country** |
| --- | --- | --- |
| AIChE Beer Brewing Competition | <https://www.aiche.org> | USA |
| America’s Cup Home Brew Contest | [https://suwaneebeerfest.com](https://suwaneebeerfest.com/) | USA |
| Best Florida Beer Homebrew Competition | [https://www.bestfloridabeer.org](https://www.bestfloridabeer.org/) | USA |
| Best of Craft Beer Awards | [https://bestofcraftbeerawards.com](https://bestofcraftbeerawards.com/) | USA |
| Brewers Cup of California | [https://brewerscupofca.com](https://brewerscupofca.com/) | USA |
| Brewski Awards | [https://www.brewskiawards.com](https://www.brewskiawards.com/) | USA |
| California Commercial Craft Beer Competition | <https://calexpostatefair.com> | USA |
| California Homebrew Competition | <https://calexpostatefair.com> | USA |
| Canada Beer Cup | [https://canadabeercup.com](https://canadabeercup.com/) | Canada |
| Canadian Brewing Awards | <https://canadianbrewingawards.com> | Canada |
| Colorado State Fair Craft Beer Competition | <https://coloradostatefair.com> | USA |
| Colorado State Fair Homebrew Competition | <https://coloradostatefair.com> | USA |
| Denver International Beer Competition | <https://denverbeercomp.com> | USA |
| Great American Beer Festival Awards | [https://www.greatamericanbeerfestival.com](https://www.greatamericanbeerfestival.com/) | USA |
| El Dorado County FAIR Homebrew and Microbrew Competitions | <https://eldoradocountyfair.org/index.html> | USA |
| Honey Beer Competition | <https://honeybeercompetition.com> | USA |
| Los Angeles International Beer Competition | [https://fairplex.com](https://fairplex.com/) | USA |
| Maryland Craft Beer Competition | <https://mdcompcup.brewingcompetitions.com/> | USA |
| Minnesota State Fair Home Brew Competition | <https://mnstatefair.brewingcompetitions.com/> | USA |
| National Homebrew Competition | [http://www.homebrewersassociation.org](http://www.homebrewersassociation.org/) | USA |
| New York International Beer Competition | <https://nyibeercompetition.com> | USA |
| New York State Craft Beer Competition | [https://www.nyscbc.com](https://www.nyscbc.com/) | USA |
| Ohio Craft Brewers Cup | <https://www.ohiocraftbrewerscup.com> | USA |
| Oregon Beer Awards | <https://www.oregonbeerawards.com> | USA |
| St. Louis Brews Microfest Homebrewing Competition | <https://stlmicrofest.org> | USA |
| Tasting Alliance Beer Competition | [https://thetastingalliance.com](https://thetastingalliance.com/) | USA |
| The United States Beer Tasting Championship | [https://www.usbtc.com](https://www.usbtc.com/) | USA |
| U.S. Open Beer Championship | [https://usopenbeer.com](https://usopenbeer.com/) | USA |
| Virginia Craft Beer Cup Competition | <https://www.virginiacraftbrewers.org> | USA |
| World Beer Competition | <https://thetastingalliance.com> | USA |
| World Beer Cup | [https://www.worldbeercup.org](https://www.worldbeercup.org/) | USA |
|  |  |  |
| **European competitions** | **Website** | **Organising country** |
| Austrian Beer Challenge | <https://bierig.org> | Austria |
| Barcelona Beer Challenge | [barcelonabeerchallenge.com](https://barcelonabeerchallenge.com/) | Spain |
| Berlin International Beer Competition | [https://www.berlininternationalbeercompetition.com](https://www.berlininternationalbeercompetition.com/) | Germany |
| Birra dell’Anno | [unionbirrai.com](http://unionbirrai.com/) | Italy |
| Brouwland Beer Competition | <https://brouwland.com/nl/> | Netherlands |
| Brussels Beer Challenge | [brusselsbeerchallenge.com](http://www.brusselsbeerchallenge.com/) | Belgium |
| Campionato Italiano Homebrewing | <https://www.movimentobirra.it> | Italy |
| Competicíon Nacional | <https://www.cerveceros-caseros.com> | Spain |
| Concours International de Lyon Beers Competition | <https://www.concourslyon.com/en> | France |
| Concours International des Produits Biologiques | <https://concoursbio.com/en> | France |
| Corso-Korsó Házidörfőzők Versenye | [https://corsokorso.brewer.hu/#](https://corsokorso.brewer.hu/) | Hungary |
| Częstochowski Konkurs Piw Domowych Bractwa Piwnego | <https://www.ebcu.org> | Poland |
| Dutch Beer Challenge | [dutchbeerchallenge.nl](http://www.dutchbeerchallenge.nl/) | Netherlands |
| European Beer Challenge | [https://europeanbeerchallenge.org](https://europeanbeerchallenge.org/) | UK |
| European Beer Star | [european-beer-star.com](http://european-beer-star.com/) | Germany |
| European Craft Beer Cup | <https://cebcexpo.eu> | Hungary |
| France Bière Challenge | [francebierechallenge.fr](https://francebierechallenge.fr/) | France |
| Frankfurt International Beer Trophy | <https://www.frankfurt-trophy.com/en/beer-trophy> | Germany |
| GBBF Homebrew Competition | <https://gbbf.org.uk> | UK |
| Golden Bohemia | <https://www.ebcu.org> | Czech Republic |
| Great British Beer Festival Homebrew Competition | <https://gbbf.org.uk> | UK |
| Greater Poland Beer Cup | <https://targipiwne.pl> | Polans |
| Házisörfőzök Nemzetközi Versenye | [http://www.elsosor.hu](http://www.elsosor.hu/) | Hungary |
| Hrvatsko Homebrew Prvenstvo | [https://pivari-grada-zagreba.hr/hhp2024/#](https://pivari-grada-zagreba.hr/hhp2024/) | Croatia |
| Independent Craft Brewers of Ireland Beer of the Year | <http://icbi.ie> | Ireland |
| International Brewing Awards | <https://www.brewingawards.org> | UK |
| International Cider Awards | <https://www.brewingawards.org> | UK |
| London Beer Competition | [https://londonbeercompetition.com](https://londonbeercompetition.com/) | UK |
| Polish Homebrewing Championships | <https://pspd.org.pl> | Poland |
| Suomen Paras Olut / Best Beer of Finland | [www.suomenparasolut.fi](http://www.suomenparasolut.fi/) | Finland |
| Swiss Beer Award | <https://swissbeeraward.ch> | Switzerland |
| The International Beer Challenge | <https://internationalbeerchallenge.com> | UK |
| Valtakunnallinen Kotiolutkilpailu | <https://olutliitto.fi> | Finland |
| World Cup of Beer | <https://www.worldcupofbeer.com> | Germany |
|  |  |  |
| **Asian competitions** | **Website** | **Organising country** |
| Asia Beer Championship | <http://asiabeerchampionship.com> | Singapore |
| Asia International Beer Competition | <https://asiainternationalbeercompetition.com> | Singapore |
|  |  |  |
| **Oceanic competitions** | **Website** | **Organising country** |
| Australian International Beer Awards | <https://www.melbourneroyal.com.au> | Australia |
| Perth Royal Beer Awards | <https://perthroyalfoodawards.raswa.org.au> | Australia |
| Royal Adelaide Beer & Cider Awards | <https://www.beerciderawards.com.au> | Australia |
| Royal Queensland Beer Awards | [https://www.rqa.com.au](https://www.rqa.com.au/) | Australia |
| Melbourne International Beer Competition | [https://melbourneinternationalbeercompetition.com](https://melbourneinternationalbeercompetition.com/) | Australia |

**Table 3.** Spirits competitions around the world (continent, website, organising country)

| **American competitions** | **Website** | **Organising country** |
| --- | --- | --- |
| American Distilling Institute International Spirits Competition | [https://distilling.com](https://distilling.com/) | USA |
| American Spirits Council Of Tasters Awards | [https://ascotawards.com](https://ascotawards.com/) | USA |
| Barleycorn Awards | <https://barleycornawards.com> | USA |
| Craft Distillers Spirits Competition | <https://www.winecompetitions.com> | USA |
| Denver International Spirits Competition | <https://denverspiritscomp.com> | USA |
| Distillers Challenge International Spirits Competition | <https://distillerschallenge.com> | USA |
| International Women’s Spirits Competiton | <https://www.winecompetitions.com> | USA |
| Latin America World Spirits Competiton | <https://thetastingalliance.com> | Mexico |
| Los Angeles International Spirits Competition | <https://fairplex.com> | USA |
| Los Angeles Invitational Wine & Spirits Challenges | <https://lainvitational.com> | USA |
| L.A. Spirits Awards | <https://www.laspiritsawards.com> | USA |
| MLSA Competition | <https://www.mlsacomp.com> | USA |
| New York International Spirits Competition | <https://www.nyispiritscompetition.com> | USA |
| New York World Wine & Spirits Competition | <https://thetastingalliance.com> | USA |
| Saint Lawrence Spirits Awards | <https://www.saintlawrencespirits.com> | USA |
| San Diego International Wine & Spirits Challenge | <https://www.sandiegowinechallenge.com> | USA |
| San Francisco Ready To Drink Competition | <https://thetastingalliance.com> | USA |
| San Francisco World Spirits Competition | <https://thetastingalliance.com> | USA |
| Spirits International Prestige Awards | <https://sipawards.com> | USA |
| Sunset International Spirits Competition | <https://sunsetcompetitions.com> | USA |
| Ultimate Beverage Challenge | <https://www.ultimate-beverage.com> | USA |
| USA Spirits Ratings | <https://usaspiritsratings.com> | USA |
| U. S. Open Whiskey & Spirits Championship | [https://usopenwhiskey.com](https://usopenwhiskey.com/) | USA |
| Wine & Spirits Wholesalers of America | [https://www.wswa.org](https://www.wswa.org/) | USA |
|  |  |  |
| **European competitions** | **Website** | **Organising country** |
| Challenge des Marques (Le Challenge des Boissons Aromatisées à base de Vin) | [https://www.challengedesmarques.com](https://www.challengedesmarques.com/) | France |
| Concours International de Lyon Spirits Competition | <https://www.concourslyon.com/en> | France |
| Concours International des Produits Biologiques | <https://concoursbio.com/en> | France |
| International Spirits Challenge | [https://internationalspiritschallenge.com](https://internationalspiritschallenge.com/internationalspiritschallenge2023/en/page/home) | UK |
| London Spirits Competition | [https://londonspiritscompetition.com](https://londonspiritscompetition.com/) | UK |
| Malt Maniacs Awards | <http://www.maltmaniacs.net/awards/> | UK |
| Pre-Mixed & RTD Masters | [https://globalspiritsmasters.com](https://globalspiritsmasters.com/) | UK |
| San Francisco World Spirits Competition – European Satellite Competiton | <https://thetastingalliance.com> | Germany |
| The Brandy Masters | [https://globalspiritsmasters.com](https://globalspiritsmasters.com/) | UK |
| The Cognac Masters | [https://globalspiritsmasters.com](https://globalspiritsmasters.com/) | UK |
| The Gin Masters | [https://globalspiritsmasters.com](https://globalspiritsmasters.com/) | UK |
| The Global Spirits Masters Competition | [https://globalspiritsmasters.com](https://globalspiritsmasters.com/) | UK |
| The Hard Seltzer Masters | [https://globalspiritsmasters.com](https://globalspiritsmasters.com/) | UK |
| The Liqueur Masters | [https://globalspiritsmasters.com](https://globalspiritsmasters.com/) | UK |
| The Low & No Masters | [https://globalspiritsmasters.com](https://globalspiritsmasters.com/) | UK |
| The Rum & Cachaça Masters | [https://globalspiritsmasters.com](https://globalspiritsmasters.com/) | UK |
| The Speciality Spirits Masters | [https://globalspiritsmasters.com](https://globalspiritsmasters.com/) | UK |
| The Tequila & Mezcal Masters | [https://globalspiritsmasters.com](https://globalspiritsmasters.com/) | UK |
| The Tonic & Mixer Masters | [https://globalspiritsmasters.com](https://globalspiritsmasters.com/) | UK |
| The Vodka Masters | [https://globalspiritsmasters.com](https://globalspiritsmasters.com/) | UK |
|  |  |  |
| **Asian competitions** | **Website** | **Szervező ország** |
| Asia International Spirits Competition | <https://asiainternationalspiritscompetition.com> | Singapore |
| Singapore World Spirits Competition | <https://thetastingalliance.com> | Singapore |
| Tokyo Whisky & Spirits Competition | <https://tokyowhiskyspiritscompetition.jp> | Japan |
|  |  |  |
| **Oceanic competitions** | **Website** | **Organising country** |
| Australian Distilled Spirits Awards | <https://www.melbourneroyal.com.au> | Australia |
| Perth Royal Distilled Spirits Awards | <https://perthroyalfoodawards.raswa.org.au> | Australia |
|  |  |  |
| **International competition** | **Website** | **Organising country** |
| International Wines and Spirits Competition | [https://iwsc.net](https://iwsc.net/) | more countries in a year |

**Table 4.** Coffee competitions around the world (continent, website, organising country)

| **American competitions** | **Website** | **Organising country** |
| --- | --- | --- |
| CoffeeFest Latte Art World Championship | <https://www.coffeefest.com/welcome> | USA |
| Cup of Excellence | [https://cupofexcellence.org](https://cupofexcellence.org/) | USA |
| U.S. Barista Competition | [https://uscoffeechampionships.org](https://uscoffeechampionships.org/) | USA |
| U.S. Brewers Competition | [https://uscoffeechampionships.org](https://uscoffeechampionships.org/) | USA |
| U.S. Coffee in Good Spirits Competition | [https://uscoffeechampionships.org](https://uscoffeechampionships.org/) | USA |
| U.S. Cup Tasters Competition | [https://uscoffeechampionships.org](https://uscoffeechampionships.org/) | USA |
| U.S. Latte Art Competition | [https://uscoffeechampionships.org](https://uscoffeechampionships.org/) | USA |
| U.S. Roasters Competition | [https://uscoffeechampionships.org](https://uscoffeechampionships.org/) | USA |
|  |  |  |
| **European competitions** | **Website** | **Organising country** |
| Coffee Masters | [https://www.londoncoffeefestival.com](https://www.londoncoffeefestival.com/) | UK |
| Dutch Brewers Cup | <https://scanederland.coffee> | Netherlands |
| Dutch Barista Championships | <https://scanederland.coffee> | Netherlands |
| Dutch Cup Tasters Championship | <https://scanederland.coffee> | Netherlands |
| International Coffee Tasting | <https://www.internationalcoffeetasting.com/ict/> | Italy |
| Irish Barista Championships | <https://linktr.ee/SCAIreland> | Ireland |
| Irish Brewers Cup | <https://linktr.ee/SCAIreland> | Ireland |
| Irish Coffee In Good Spirits | <https://linktr.ee/SCAIreland> | Ireland |
| Irish Cupping Tasters Championship | <https://linktr.ee/SCAIreland> | Ireland |
| Irish Latte Art Championships | <https://linktr.ee/SCAIreland> | Ireland |
| Polish Barista Championship | <https://scap.pl> | Poland |
| Polish Brewers Cup | <https://scap.pl> | Poland |
| Polish Cezve/Ibrik | <https://scap.pl> | Poland |
| Polish Coffee In Good Spirits Championship | <https://scap.pl> | Poland |
| Polish Cup Tasters Championship | <https://scap.pl> | Poland |
| Polish Latte Art Championship | <https://scap.pl> | Poland |
| Polish Roasting Championship | <https://scap.pl> | Poland |
| UK Barista Championship | <https://scauk.coffee> | UK |
| UK Brewers Cup | <https://scauk.coffee> | UK |
| UK Coffee in Good Spirits Championship | <https://scauk.coffee> | UK |
| UK Coffee Roasters | <https://scauk.coffee> | UK |
| UK Cup Tasters Championship | <https://scauk.coffee> | UK |
| UK Latte Art Championship | <https://scauk.coffee> | UK |
| World Coffee Challenge | [https://www.worldcoffeechallenge.com](https://www.worldcoffeechallenge.com/) | Spain |
|  |  |  |
| **Asian competitions** | **Website** | **Organising country** |
| Japan Barista Championship | <https://scaj.org> | Japan |
| Japan Brewers Cup | <https://scaj.org> | Japan |
| Japan Coffee in Good Spirits Championship | <https://scaj.org> | Japan |
| Japan Coffee Roasting Championship | <https://scaj.org> | Japan |
| Japan Cup Tasters Championship | <https://scaj.org> | Japan |
| Japan Latte Art Championship | <https://scaj.org> | Japan |
| Japan Siphonist Championship | <https://scaj.org> | Japan |
| Korea Barista Championship | <https://www.cafeshow.com> | Korea |
| Korea Team Barista Championship | <https://www.cafeshow.com> | Korea |
| Malaysia National Coffee Championship | <https://msca.org.my> | Malaysia |
| Master of Brewing | <https://www.cafeshow.com> | Korea |
| Master of Cupping | <https://www.cafeshow.com> | Korea |
| Philippine National Barista Competition | [https://philippinenationalcoffeecompetition.com](https://philippinenationalcoffeecompetition.com/) | Republic of the Philippines |
| Philippine National Brewers Cup | [https://philippinenationalcoffeecompetition.com](https://philippinenationalcoffeecompetition.com/) | Republic of the Philippines |
| Philippine National Cup Tasters | [https://philippinenationalcoffeecompetition.com](https://philippinenationalcoffeecompetition.com/) | Republic of the Philippines |
| Philippine National Latte Art Championship | [https://philippinenationalcoffeecompetition.com](https://philippinenationalcoffeecompetition.com/) | Republic of the Philippines |
| Singapore National Barista Championship | <https://food2go.asia> | Singapore |
| Singapore National Brewers Cup | <https://food2go.asia> | Singapore |
| World Latte Art Battle | <https://www.cafeshow.com> | Korea |
| World Siphonist Championship | <https://scaj.org> | Japan |
|  |  |  |
| **Oceanic competitions** | **Website** | **Organising country** |
| ASCA Brewers Cup and Cup Tasters | <https://australianspecialtycoffee.com.au> | Australia |
| ASCA Coffee in Good Spirits Championship | <https://australianspecialtycoffee.com.au> | Australia |
| ASCA Coffee Roasting Championship | <https://australianspecialtycoffee.com.au> | Australia |
| Australian International Coffee Awards | <https://www.melbourneroyal.com.au> | Australia |
|  |  |  |
| **International competitions** | **Website** | **Organising country** |
| World AeroPress Championship | [https://worldaeropresschampionship.com](https://worldaeropresschampionship.com/) | different country in every year |
| World of Coffee | [https://www.worldofcoffee.org](https://www.worldofcoffee.org/) | more countries in a year |
| World Barista Championship | [https://worldcoffeeevents.org](https://worldcoffeeevents.org/) | more countries in a year |
| World Brewers Cup | [https://worldcoffeeevents.org](https://worldcoffeeevents.org/) | more countries in a year |
| World Cezve/Ibrik Championship | [https://worldcoffeeevents.org](https://worldcoffeeevents.org/) | more countries in a year |
| World Coffee in Good Spirits Championship | [https://worldcoffeeevents.org](https://worldcoffeeevents.org/) | more countries in a year |
| World Coffee Roasting Championship | [https://worldcoffeeevents.org](https://worldcoffeeevents.org/) | more countries in a year |
| World Cup Tasters Championship | [https://worldcoffeeevents.org](https://worldcoffeeevents.org/) | more countries in a year |
| World Latte Art Championship | [https://worldcoffeeevents.org](https://worldcoffeeevents.org/) | more countries in a year |

**Table 5.** Whiskey competitions around the world (continent, website, organising country)

| **American competitions** | **Website** | **Organising country** |
| --- | --- | --- |
| Canadian Whisky Awards | <https://canadianwhiskyawards.com> | Canada |
| Heartland Whiskey Competition | [https://heartlandwhiskeycompetition.com](https://heartlandwhiskeycompetition.com/) | USA |
| International Whisky Competition | [https://www.whiskycompetition.com](https://www.whiskycompetition.com/) | USA |
| Jim Murray's Whisky Bible Awards | [https://www.whiskybible.com](https://www.whiskybible.com/) | USA |
| North American Bourbon and Whiskey Competition | [https://whiskeycomp.com](https://whiskeycomp.com/) | USA |
| Seattle World Whiskey Day | <https://seattleworldwhiskyday.com> | USA |
| Scottish Whisky Competitions | <https://scottishwhiskycompetitions.com> | USA |
| The American Whiskey Masters | [https://globalspiritsmasters.com](https://globalspiritsmasters.com/) | USA |
| U. S. Open Whiskey & Spirits Championship | [https://usopenwhiskey.com](https://usopenwhiskey.com/) | USA |
| Whiskies of the World Awards | <https://www.whiskiesoftheworld.com> | USA |
|  |  |  |
| **European competitions** | **Website** | **Organising country** |
| Luxury & Rare Whisky Competitions | [https://luxuryandrarewhisky.co.uk](https://luxuryandrarewhisky.co.uk/) | Scotland |
| Malt Maniacs Awards | <http://www.maltmaniacs.net> | UK |
| The Irish Whiskey Masters | <https://globalspiritsmasters.com> | UK |
| The Scotch Whisky Masters | <https://globalspiritsmasters.com> | UK |
| The World Whisky Masters | <https://globalspiritsmasters.com> | UK |
| World Whiskies Awards | [https://www.worldwhiskiesawards.com](https://www.worldwhiskiesawards.com/) | UK |
|  |  |  |
| **Asian competition** | **Website** | **Organising country** |
| Tokyo Whisky & Spirits Competition | <https://tokyowhiskyspiritscompetition.jp> | Japan |
|  |  |  |
| **Oceanic competition** | **Website** | **Organising country** |
| Australian Malt Whisky Tasting Championship | [https://www.amwtc-smws.com](https://www.amwtc-smws.com/) | Australia |

**Table 6.** Water competitions around the world (continent, website, organising country)

| **American competitions** | **Website** | **Organising country** |
| --- | --- | --- |
| Berkeley Springs International Water Tasting | [https://berkeleyspringswatertasting.com](https://berkeleyspringswatertasting.com/) | USA |
| National Rural Water Association’s Great American Water Taste Test | <https://nrwa.org/great-american-water-taste-test/> | USA |
| Northeastern Ontario Water Works Conference Drinking Water Taste Test Competition | [https://www.neowwc.com](https://www.neowwc.com/) | USA |
|  |  |  |
| **European competition** | **Website** | **Organising country** |
| Golden World Spring | [https://www.goldenspring.cz](https://www.goldenspring.cz/) | Czech Republic |
|  |  |  |
| **Asian competition** | **Website** | **Organising country** |
| Guangzhou International Water Tasting Competition | <http://www.ihe-china.com/en/> | China |
|  |  |  |
| **International competition** | **Website** | **Organising country** |
| FineWaters Taste & Design Awards | <https://finewaters.com> | different country in every year |

**Table 7.** Pálinka competitions around the world (continent, website, organising country)

| **European competitions** | **Website** | **Organising country** |
| --- | --- | --- |
| Baráti Pálinkaverseny | <https://palinkapont.hu> | Slovakia |
| Beremendi Pálinkamustra | <https://palinkapont.hu> | Hungary |
| Blisseva Nemzetközi Pálinka és Párlatverseny | <https://palinkapont.hu> | Romania |
| Brillante Nemzetközi Pálinka- és Párlatverseny | <https://gyulaipalinkafesztival.hu/en/> | Hungary |
| Derecskei Pálinkamustra | <https://palinkapont.hu> | Hungary |
| Destillata | [https://destillata.at](https://destillata.at/) | Austria |
| Enyingi Pálinkamustra | <https://palinkapont.hu> | Hungary |
| Erdei és Vadon Termő Gyümölcsökből Készült Pálinkák Országos Versenye | <https://palinkapont.hu> | Hungary |
| Erdélyi pálinka és párlatverseny | <https://palinkapont.hu> | Romania |
| Etei körzeti Pálinka és Párlatverseny | <https://palinkapont.hu> | Hungary |
| Gombai Pálinka és Párlatverseny | <https://palinkapont.hu> | Hungary |
| Gyermelyi Pálinka Est és Pálinkás Programok | <https://palinkapont.hu> | Hungary |
| HunDeszt the Pálinka Competition’s | <https://www.dunaihajospalinka.hu/en/palinkaversenyek> | Hungary |
| Jáki Pálinkaverseny | <https://palinkapont.hu> | Hungary |
| Jász Világtalálkozó Pálinkamustra és Párlatverseny | <https://palinkapont.hu> | Hungary |
| Jászkiséri Nemzetközi Pálinka- és Párlatverseny | <https://palinkapont.hu> | Hungary |
| Jászsági Pálinkamustra és Párlatverseny | <https://palinkapont.hu> | Hungary |
| Jubileumi Pálinka és Párlatverseny | <https://palinkapont.hu> | Hungary |
| Kárászi Pálinka- és Nemzetközi Párlatverseny | <https://palinkapont.hu> | Hungary |
| Kiskunlacházi Párlatverseny | <https://palinkapont.hu> | Hungary |
| Kisüsti Pálinkaverseny | <https://palinkapont.hu> | Hungary |
| Magnus Spirituum Pálinka- és Párlatverseny | <https://palinkapont.hu> | Hungary |
| Nemzetközi Gönci Pálinkamustra | <https://palinkapont.hu> | Hungary |
| Nemzetközi Pálinkamustra | <https://palinkapont.hu> | Hungary |
| Nemzetközi pálinkaverseny | <https://palinkapont.hu> | Serbia |
| Ormánsági Pálinkaverseny | <https://palinkapont.hu> | Hungary |
| Országos és nemzetközi pálinka – gyümölcspárlat, Duna Arany Párlata | <https://palinkapont.hu> | Slovakia |
| Országos Pálinka- és Törkölypálinka Verseny | <https://palinkapont.hu> | Hungary |
| Pálinkamustra | <https://palinkapont.hu> | Hungary |
| Pannónia Párlatverseny | <https://palinkapont.hu> | Hungary |
| QUINTESSENCE Pálinka- és Párlatverseny | <https://quintessence-palinka.hu/hu/> | Hungary |
| Regionális Palóc Pálinka- és Párlatverseny | <https://palinkapont.hu> | Hungary |
| Sajóbábonyi Regionális Pálinkaverseny | <https://palinkapont.hu> | Hungary |
| Sárisápi Nyílt Pálinka- és Párlatverseny | <https://palinkapont.hu> | Hungary |
| Sárréti Pálinka és Párlatmustra | <https://palinkapont.hu> | Hungary |
| Scientia Master Spirit Competition | [https://scientiaegyesulet.hu](https://scientiaegyesulet.hu/) | Hungary |
| Sólyi Nemzetközi Pálinka- és párlatverseny | <https://palinkapont.hu> | Hungary |
| Somogy Vármegyei Bor- és Pálinkaverseny | <https://palinkapont.hu> | Hungary |
| Vajdasági és Temerini Pálinkaverseny | <https://palinkapont.hu> | Serbia |
| Vas Megyei Nyílt Pálinka- és párlatverseny | <https://palinkapont.hu> | Hungary |
| Vecsési Pálinka Verseny | <https://palinkapont.hu> | Hungary |
| World Spirits Award | <https://www.world-spirits.com/en/> | Austria |

**Table 8.** Champagne competitions around the world (continent, website, organising country)

| **European competitions** | **Website** | **Organising country** |
| --- | --- | --- |
| Concours Mondial de Bruxelles | <https://concoursmondial.com/fr/> | Belgium |
| Glass of Bubbly Awards | <https://glassofbubbly.com> | UK |
| The Champagne Club Awards | <https://www.champagneclub.com> | France |
| The Champagne Masters | [https://www.globalwinemasters.com](https://www.globalwinemasters.com/) | UK |
| The Champagne & Sparkling Wine World Championships | [https://www.champagnesparklingwwc.co.uk](https://www.champagnesparklingwwc.co.uk/) | UK |
